# An ANGPTL8‐AKT2‐mTOR Axis Drives Adipose Senescence and Aging‐Related Functional Decline

**DOI:** 10.1111/acel.70671

**Published:** 2026-08-16

**Authors:** Yi He, Limeng Pan, WenJun Ping, Chen Meng, Xiaoyu Meng, Yaming Guo, Ranran Kan, Yuxi Xiang, Beibei Mao, Siyi Wang, Danpei Li, Xuefeng Yu

**Affiliations:** ^1^ Department of Endocrinology Tongji Hospital, Tongji Medical College, Huazhong University of Science and Technology Wuhan Hubei China; ^2^ Hubei Provincial Clinical Medical Research Center for Endocrinology and Metabolic Diseases Wuhan Hubei China; ^3^ Department of Metabolic and Bariatric Surgery The Third Xiangya Hospital, Central South University Changsha China; ^4^ Department of Joint Bone Disease Surgery Changhai Hospital, Naval Medical University Shanghai China

**Keywords:** adipose tissue, aging, aging clock, AKT–mTOR signaling, ANGPTL8, cellular senescence, mortality prediction

## Abstract

Adipose tissue senescence is increasingly recognized as a key driver of systemic aging and age‐related functional decline, yet the endocrine regulators that actively promote this process remain poorly defined. Angiopoietin‐like protein 8 (ANGPTL8) is a metabolic factor implicated in lipid metabolism and inflammation and has been associated with multiple aging‐related disorders. However, its direct role in adipose tissue senescence and organismal aging remains unclear. Here, we identify ANGPTL8 as a previously unrecognized regulator of adipose tissue aging through integrative analyzes of human cohorts, animal models, transcriptomics, and cellular studies. In a large human cohort, circulating ANGPTL8 levels were strongly associated with biological aging and mortality risk and significantly improved machine learning–based models for age and survival prediction. Consistent with these findings, genetic deletion of *Angptl8* in mice extended lifespan, attenuated aging‐associated functional decline, and reduced senescence markers in adipose tissue. Transcriptomic analyzes revealed age‐dependent upregulation of ANGPTL8 in adipocytes accompanied by activation of pro‐senescent transcriptional programs. Mechanistically, ANGPTL8 directly interacted with AKT2 and activated the AKT–mTOR–S6K signaling pathway, thereby promoting cell‐autonomous adipocyte senescence. Genetic or pharmacological inhibition of this pathway abolished the pro‐senescent effects of ANGPTL8. Collectively, our findings identify ANGPTL8 as an endocrine regulator linking metabolic dysfunction to adipose tissue senescence and systemic aging, highlighting the ANGPTL8–AKT2–mTOR axis as a potential therapeutic target for delaying age‐associated functional decline.

## Graphical abstract

ANGPTL8 increases with age in humans and mice and serves as a key predictor of biological aging and mortality. Adipocyte‐derived ANGPTL8 promotes cellular senescence through AKT2–mTOR signaling, whereas genetic deletion of *Angptl8* attenuates adipose tissue aging and extends lifespan.

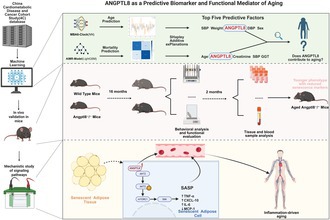

## 1 Introduction

Aging is a complex and tightly regulated biological process characterized by a progressive decline in cellular and tissue function, ultimately increasing vulnerability to chronic diseases and functional impairment (López‐Otín et al. 2023). Increasing evidence indicates that aging is accompanied by a sustained state of low‐grade, sterile inflammation—commonly termed inflammaging—rather than representing a purely passive accumulation of molecular damage (Ferrucci and Fabbri 2018). A central driver of this process is the age‐dependent accumulation of senescent cells, which secrete a diverse repertoire of pro‐inflammatory cytokines, chemokines, growth factors, and proteases collectively known as the senescence‐associated secretory phenotype (SASP) (Campisi and d'Adda di Fagagna 2007; Franceschi et al. 2000; Franceschi et al. 2018). Through the establishment of a self‐reinforcing inflammatory microenvironment (Behmoaras and Gil 2021), SASP signaling disrupts tissue homeostasis, propagates cellular dysfunction across organs, and accelerates systemic aging, thereby predisposing organisms to a broad spectrum of age‐associated disorders, including metabolic, neurodegenerative, and cardiometabolic diseases (Coppé et al. 2010). In the context of a rapidly aging global population (Partridge et al. 2018), elucidating molecular mediators that link cellular senescence to systemic inflammation represents a critical prerequisite for the development of strategies aimed at delaying aging and extending health span.

Among peripheral tissues implicated in systemic inflammaging, adipose tissue has emerged as a central integrative hub linking metabolic regulation to inflammatory signaling (Hotamisligil 2017). Beyond its classical role as an energy storage depot, adipose tissue functions as a highly dynamic endocrine organ that exerts broad regulatory effects on whole‐body metabolic and immune homeostasis (Ou et al. 2022). Aging is accompanied by profound structural and functional remodeling of adipose tissue, including ectopic lipid accumulation, dysregulated adipokine secretion, impaired insulin sensitivity, and heightened inflammatory signaling (Tchkonia et al. 2010).

Notably, the age‐associated accumulation of senescent adipocytes and adipose progenitor cells amplifies both local and systemic inflammation through the sustained release of SASP factors (Dahlquist and Camell 2022). This chronic inflammatory output not only disrupts adipose tissue homeostasis but also propagates inflammatory cues to distal organs, thereby positioning adipose tissue as an active driver—rather than a passive target—of aging‐associated metabolic dysfunction (Franceschi et al. 2017). Given its endocrine prominence and systemic reach, identifying adipose‐derived molecular mediators that couple tissue senescence to organismal aging is therefore of particular importance.

Within this context, a critical unanswered question is which adipose‐derived endocrine factors mechanistically couple adipose tissue senescence to systemic inflammation and organismal aging. Angiopoietin‐like protein 8 (ANGPTL8) is a secreted metabolic regulator predominantly expressed in the liver and adipose tissue and belongs to the angiopoietin‐like protein family (Abu‐Farha et al. 2020). While ANGPTL8 has been extensively studied for its role in lipid metabolism and energy homeostasis, emerging evidence suggests that it may also exert immunometabolism functions under conditions of metabolic stress and inflammation (Ye et al. 2023; Zhang et al. 2023).

Importantly, our group has previously demonstrated that circulating ANGPTL8 levels are significantly elevated across multiple age‐associated pathological conditions, including diabetic nephropathy (Pan et al. 2026), non‐alcoholic fatty liver disease (Li et al. 2023), diabetes‐related cognitive impairment (Meng et al. 2024), and skeletal aging (Guo et al. 2025). Beyond our findings, multiple independent investigations have linked increased ANGPTL8 levels to cardiovascular diseases, highlighting a shared association between ANGPTL8 dysregulation and age‐related cardiometabolic disorders (Jiao et al. 2023; Morinaga et al. 2023; Silbernagel et al. 2025; Ye et al. 2026). More recently, ANGPTL8 has also been implicated in aging‐related pathological processes beyond metabolic tissues. In Alzheimer's disease models, ANGPTL8 deficiency was shown to attenuate neuroinflammation and improve cognitive function, suggesting that ANGPTL8 may contribute to chronic inflammation and tissue dysfunction during aging (Wei et al. 2025). Although clinically distinct, these disorders share common underlying features, namely chronic low‐grade inflammation, metabolic dysregulation, and progressive functional decline—hallmarks that closely overlap with fundamental biological processes of aging. In line with this notion, our previous population‐based analysis in individuals with diabetes demonstrated that elevated circulating ANGPTL8 levels were significantly associated with increased all‐cause mortality (Zou et al. 2020), underscoring its potential relevance to aging‐related outcomes in humans. The consistent upregulation of ANGPTL8 across this spectrum of aging‐related diseases suggests that its involvement may extend beyond serving as a secondary biomarker of metabolic imbalance. Instead, these observations raise the possibility that ANGPTL8 functions as an active molecular mediator linking metabolic dysfunction to inflammation‐driven aging remodeling. Nevertheless, whether ANGPTL8 plays a causal role in driving adipose tissue senescence and systemic aging, and the molecular mechanisms through which it may exert such effects, remain largely unexplored—prompting the present study.

To systematically interrogate the role of ANGPTL8 in aging, we established an integrative, multi‐scale research framework that combines population‐level modeling with experimental validation in animal and cellular systems. Leveraging comprehensive clinical and biochemical data from the China Cardiometabolic Disease and Cancer Cohort (4C) Study, we first employed machine learning–based approaches to construct two complementary predictive models: the Metabolic Biomarker and ANGPTL8‐Based Aging Clock (MBA8‐Clock), a multi‐biomarker aging clock integrating circulating ANGPTL8 to estimate biological age, and the ANGPTL8‐Integrated Mortality Risk Model (AIMR‐Model), an ANGPTL8‐informed framework designed to predict 10‐year all‐cause mortality. These models enabled an unbiased evaluation of the associations between ANGPTL8, biological aging, and long‐term mortality at the population level.

Building upon these epidemiological observations, we next employed genetic loss‐of‐function approaches together with reductionist cellular systems to determine whether ANGPTL8 causally contributes to aging‐related phenotypes. Using *Angptl8* knockout mice and adipocyte‐based in vitro models, we examined aging‐associated functional decline, adipose tissue remodeling, and molecular signatures of cellular senescence. Finally, we explored the signaling mechanisms through which ANGPTL8 regulates adipocyte senescence, focusing particularly on the AKT/mTOR/S6K signaling pathway and cell‐intrinsic molecular networks that connect metabolic regulation with aging processes. Through this integrative strategy, our study moves from epidemiological association to experimental causality and molecular mechanism, thereby providing a comprehensive framework for understanding the role of ANGPTL8 in adipose tissue aging and systemic aging.

## 2 Methods

### 2.1 Data Source and Ethics Statement

Clinical data were derived from the China Cardiometabolic Disease and Cancer Cohort (4C) Study, which was conducted using standardized protocols and predesigned case report forms. The study involving human participants was approved by the Ethics Committees of Ruijin Hospital, Shanghai Jiao Tong University School of Medicine (Approval No. 14/2004), and Tongji Hospital, Tongji Medical College, Huazhong University of Science and Technology (Approval No. TJ‐IRB20231125). Written informed consent was obtained from all participants prior to enrollment, in accordance with the principles of the Declaration of Helsinki.

All animal experiments were conducted in compliance with institutional and national regulations for the care and use of laboratory animals. The experimental protocol was reviewed and approved by the Institutional Animal Care and Use Committee (IACUC) of the Institute of Model Animals, Tongji Hospital, Huazhong University of Science and Technology (Approval No. TJH‐202303011). All procedures adhered to the National Institutes of Health (NIH) Guide for the Care and Use of Laboratory Animals.

### 2.2 Missing Data Handling

The extent of missing data in the China Cardiometabolic Disease and Cancer Cohort (4C) Study is summarized in Table S1. To preserve statistical power and minimize imputation bias, missing values were estimated using the k‐nearest neighbor (KNN) algorithm with *k* = 10.

After imputation, the dataset was randomly partitioned into training (70%) and validation (30%) cohorts. The 4C cohort included 9904 participants (median age = 60.3 years; interquartile range [IQR], 53–68 years), of whom 6426 (64.9%) were women. During a median follow‐up of 10 years, 1441 deaths (14.5%) were documented. The cohort encompassed a wide range of age and metabolic health profiles, providing a representative sample of the general population.

KNN imputation was applied to selected metabolic and biochemical variables, including: glycated hemoglobin (HbA1c; 0.19%), fasting blood glucose (FBG; 3.25%), postprandial blood glucose (PBG; 4.35%), creatinine, low‐density lipoprotein cholesterol (LDL‐C), γ‐glutamyl transferase (GGT), and waist‐to‐hip ratio (WHR; 0.55% each), high‐density lipoprotein cholesterol (HDL‐C) and triglycerides (TG; 0.52% each), total cholesterol (TC), alanine aminotransferase (ALT), aspartate aminotransferase (AST), and waist circumference (0.50% each), as well as body mass index (BMI), height, and weight (0.25% each).

Of all participants, 6933 were assigned to the training cohort and 2971 to the validation cohort. Circulating ANGPTL8 levels were available for 3637 individuals, forming the A8 sub‐cohort, which was similarly divided into training and validation sets in a 7:3 ratio. Baseline characteristics and mortality outcomes were well balanced between training and validation cohorts in both datasets.

Across both the full cohort and the ANGPTL8 sub‐cohort, participants who died during follow‐up were generally older and exhibited higher levels of creatinine, systolic blood pressure, and fasting glucose compared with survivors, consistent with established mortality risk profiles.

### 2.3 Feature Selection Strategy

Feature selection was performed exclusively within the training cohort to prevent data leakage and ensure unbiased model development.

Pairwise Pearson correlation analysis was first conducted to assess multicollinearity among candidate predictors, applying a conservative threshold of *r* > 0.85. When strong correlations were identified, the variable with higher clinical interpretability or biological relevance was retained.

To identify the most informative predictors, two complementary approaches were implemented. The Boruta algorithm, based on random forest classification, evaluates the importance of each feature by iteratively comparing it with randomly permuted “shadow” variables, thereby isolating truly relevant predictors. In parallel, Least Absolute Shrinkage and Selection Operator (LASSO) regression was applied to perform variable shrinkage and selection via L1 regularization, balancing model sparsity and predictive power.

The intersection of features identified by both Boruta and LASSO was defined as the final predictor set, capturing variables robust under both linear and nonlinear relationships. This integrative approach improves feature stability, interpretability, and generalizability across machine learning models.

As shown in Figure S1A, no variable pairs exceeded the multicollinearity threshold, and all were retained for feature selection. The combined Boruta–LASSO procedure identified 22 key predictors, including ANGPTL8, HbA1c, Insulin, FBG, PBG, Creatinine, HDL‐C, LDL‐C, TC, TG, ALT, AST, GGT, SBP, DBP, Pulse, Weight, Waist circumference, Hip circumference, Snore, Smoking, and Exercise (Figure S1B–D). These predictors were subsequently used for model construction using five machine learning classifiers: Random Forest, Neural Network, LightGBM, Multilayer Perceptron, and XGBoost.

### 2.4 Frailty Index and Metabolic Phenotype Assessment

Frailty index (FI) was calculated according to the deficit accumulation approach. Each deficit was dichotomized or mapped into the 0.00–1.00 interval, with 0.00 indicating the absence of a deficit (the healthiest state) and 1.00 indicating the maximal expression of the deficit (the unhealthiest state). The frailty index for each participant was calculated as the number of deficits present divided by the total number of deficits considered. A total of 28 health deficits were included in the FI calculation, and detailed descriptions of these variables are provided in Supplementary Table S6.

To evaluate visceral adiposity‐related metabolic dysfunction, the Metabolic Score for Visceral Fat (METS‐VF) was calculated as previously described. The formula used was:

$$\text{METS}-\mathrm{VF}=4.466+0.011\times {\left[\ln\left(\text{METS}-\mathrm{IR}\right)\right]}^{3}+3.239\times {\left[\ln\left(\text{WHtR}\right)\right]}^{3}+0.319\times \mathrm{sex}+0.594\times \ln\left(\mathrm{age}\right),$$

where METS‐IR is the metabolic score for insulin resistance, WHtR is the waist‐to‐height ratio, sex was coded as 1 for males and 0 for females, and age was expressed in years.

### 2.5 μCT and Histomorphometric Analysis

The femurs were subjected to high‐resolution micro–computed tomography (μCT) using a NemoMicroCT system (nmc‐200, China) to assess bone microarchitecture. Scanning was performed at an isotropic voxel size of 35 μm, with an X‐ray source voltage of 60 kV and a current of 0.13 mA. The acquired images were reconstructed using Recon to generate three‐dimensional representations of the bone structure.

### 2.6 Magnetic Resonance Imaging (MRI) and Adipose Tissue Quantification

Whole‐body magnetic resonance imaging (MRI) using a 9.4‐T small‐animal MRI system (uMR 9.4 T, United Imaging Life Science Instrument, Shanghai, China) equipped with an 86‐mm volume transmit/receive coil (Volume Coil‐86). Mice were anesthetized with isoflurane and positioned in the prone position throughout image acquisition.

T2‐weighted fast spin‐echo (FSE‐2D‐T2WI) and water‐fat imaging (FSE‐WFI) sequences were acquired for whole‐body fat assessment. For the FSE‐2D‐T2WI sequence, the imaging parameters were as follows: repetition time (TR) = 300 ms, echo time (TE) = 26.76 ms, field of view (FOV) = 46 × 53 mm, matrix size = 153 × 176, slice thickness = 1.0 mm, 28 contiguous slices, bandwidth = 280 Hz/pixel, echo train length (ETL) = 11, voxel size = 0.3 × 0.3 × 1.0 mm^3^, and number of excitations (NEX) = 8. For the FSE‐WFI sequence, the parameters were: TR = 3000 ms, TE = 46.06 ms, FOV = 46 × 96 mm, matrix size = 153 × 320, slice thickness = 1.0 mm, 28 contiguous slices, bandwidth = 300 Hz/pixel, ETL = 15, voxel size = 0.3 × 0.3 × 1.0 mm^3^, and NEX = 8.

Because of the large body size of aged mice, the upper and lower body regions were scanned separately using identical acquisition parameters and subsequently merged to generate complete whole‐body images. Visceral adipose tissue (VAT) and subcutaneous adipose tissue (SAT) were identified on fat‐sensitive images according to their anatomical locations. Fat areas were manually segmented on each slice, and total adipose tissue volumes were calculated by summing the segmented areas across all slices and multiplying by slice thickness. The VAT/SAT ratio was subsequently calculated to evaluate age‐associated alterations in body fat distribution.

### 2.7 Lifespan Monitoring and Survival Analysis

Mice were monitored longitudinally throughout aging until spontaneous death or the predefined humane endpoint was reached. Humane endpoint criteria were established according to institutional animal welfare guidelines and included severe age‐associated deterioration, such as inability to access food or water, severe loss of body condition, or other signs of distress (e.g., severe rectal prolapse). Animals reaching humane endpoints were humanely euthanized, and the date of euthanasia was recorded as the survival endpoint. All deceased animals underwent postmortem examination to exclude non‐aging‐related causes of death, such as severe injury, infection, or other pathological conditions unrelated to physiological aging. Kaplan–Meier survival curves were generated, and differences in survival distributions between genotypes were assessed using the log‐rank (Mantel–Cox) test. No censoring was applied because all animals reached a defined survival endpoint.

### 2.8 Open Field Test (OFT)

To assess locomotor activity and exploratory behavior in a novel environment, the Open Field (OF) apparatus was used. The arena consisted of a dark‐colored acrylic box (48 × 48 cm). Lighting was set to 80 lx in the center (measured at the square center) and 50–60 lx at the corners. A central area (16 × 16 cm) was defined at the center of the arena. Animals were habituated to the laboratory environment 1 day prior to testing. For the OFT, animals were placed in the center of the arena, and the experimenter exited the room while a camera positioned above the arena recorded the animal's movements for a duration of 30 min. The “Viewer” software (Biobserve GmbH, Germany) was used to analyze the distance traveled and the time spent in the central area. Throughout the open field test, the distance traveled and the number of grooming behaviors were measured as indices of locomotor activity. The time spent immobile and in the periphery of the arena was considered as proxies for anxiety levels.

### 2.9 Rotarod Test for Assessing Motor Coordination

Motor coordination was assessed using an automated rotarod apparatus (YLS‐4C, Yiyan Tech) according to a standardized protocol. Prior to formal testing, mice underwent a 72‐h acclimation period that included daily training trials. Each mouse was placed in a designated compartment on the rotating rod, which accelerated linearly from an initial speed of 4 rpm to a maximum of 44 rpm at a rate of 8 rpm/min. During the training phase, the session was terminated once the mouse fell off the rod three consecutive times. On the fourth day, formal testing was conducted, and each mouse was subjected to a single trial. The latency to fall (i.e., the duration the mouse remained on the rotating rod) was recorded as a measure of motor coordination and balance.

### 2.10 Immunofluorescence (IF) Staining

Tissues or cells were fixed in 4% paraformaldehyde (PFA) for 15–30 min at room temperature, followed by permeabilization with 0.1% Triton X‐100 in phosphate‐buffered saline (PBS) for 10 min. After permeabilization, the samples were blocked with 5% normal goat circulating or BSA in PBS for 1 h at room temperature to reduce non‐specific binding. The primary antibody was diluted in blocking solution and incubated with the samples overnight at 4°C. After washing with PBS, the samples were incubated with the appropriate fluorophore‐conjugated secondary antibody (e.g., Alexa Fluor‐conjugated antibodies) for 1 h at room temperature in the dark. Nuclei were stained with DAPI (1 μg/mL) for 5–10 min. The samples were washed again with PBS and mounted using a mounting medium with anti‐fade reagent. Fluorescent images were captured using a confocal or fluorescence microscope. The fluorescence intensity was quantified using ImageJ software.

### 2.11 β‐Galactosidase Staining (Senescence‐Associated β‐Galactosidase Staining)

Senescence‐associated β‐galactosidase (SA‐β‐gal) staining was performed in both cultured cells and adipose tissue cryosections. Cultured cells were fixed in 4% paraformaldehyde (PFA) for 10–15 min at room temperature. Adipose tissues were embedded in OCT compound, snap‐frozen, and sectioned at a thickness of 8–10 μm using a cryostat. Tissue cryosections were fixed in 4% PFA for 10 min at room temperature.

After fixation, samples were washed with PBS and incubated with staining solution containing 1 mg/mL X‐gal (5‐bromo‐4‐chloro‐3‐indolyl‐β‐D‐galactopyranoside) in 40 mM citrate buffer (pH 6.0), 150 mM NaCl, 2 mM MgCl_2_, and 5 mM potassium ferrocyanide for 12–24 h at 37°C in the absence of CO_2_ and protected from light. Fresh staining solution was applied when necessary. The reaction was terminated by washing with PBS. Images were captured using a light microscope. Positive SA‐β‐gal staining was identified by the presence of blue staining within cells or tissue sections.

### 2.12 Isolation of Adipose Stromal Vascular Fraction, Primary Preadipocyte Culture, and Adipocyte Differentiation

The stromal vascular fraction (SVF), containing preadipocytes derived from subcutaneous white adipose tissue (SAT), was isolated from 6 to 8‐week‐old mice as previously described. Briefly, SAT depots were harvested under sterile conditions and immediately placed in cold phosphate‐buffered saline (PBS) supplemented with 1% antibiotic/antimycotic (penicillin, streptomycin, and amphotericin B) for further processing. The SAT was then minced into small pieces and digested with Type II collagenase (Cat# C6885, Sigma) in Krebs‐Ringer Bicarbonate Hepes (KRBH, pH 7.4) buffer containing 2% defatted BSA at 37°C for 30 min with gentle agitation. After digestion, the tissue was filtered through a 70 μm cell strainer to remove large debris. The filtrate was washed with KRBH and centrifuged at 4°C for 10 min at 300 g to isolate the SVF. The SVF, which contains preadipocytes, was cultured in DMEM supplemented with 10% fetal bovine serum (FBS), 1% penicillin/streptomycin (P/S), 1% amphotericin B, and 16 μM biotin (regular medium) until confluence.

For adipogenic differentiation, confluent primary preadipocytes were maintained in differentiation induction medium consisting of DMEM supplemented with 10% fetal bovine serum (FBS), 1% penicillin/streptomycin (P/S), 0.5 mM 3‐isobutyl‐1‐methylxanthine (IBMX; Sigma), 1 μM dexamethasone (Sigma), 5 μg/mL insulin (Sigma), and 1 μM rosiglitazone (Sigma). Cells were cultured in induction medium for 48 h, after which the medium was replaced with maintenance medium containing DMEM supplemented with 10% FBS, 1% P/S, 5 μg/mL insulin, and 1 μM rosiglitazone. The maintenance medium was refreshed every 2 days throughout the differentiation period. After 8–10 days of induction, the majority of cells exhibited characteristic lipid droplet accumulation, indicating successful differentiation into mature adipocytes.

### 2.13 RNA Isolation and Real‐Time Quantitative PCR (RT‐qPCR)

Total RNA was extracted using TRIzol reagent (Thermo Fisher Scientific, MA, USA, Cat# 15596018) according to the manufacturer's instructions and quantified using a Nanodrop spectrophotometer. For cDNA synthesis, 1 μg of total RNA was reverse transcribed using Hifair II 1st Strand cDNA Synthesis SuperMix (Yeasen, Shanghai, China, Cat#11120ES60). RT‐qPCR was performed using the Hieff UNICON qPCR SYBR Green Master Mix (Low Rox) (Yeasen, Shanghai, China, Cat#11199ES08) under the following conditions: initial denaturation at 95°C for 30 s, followed by 40 cycles of 95°C for 10 s, and 60°C for 30 s. Each sample was analyzed in triplicate. Relative mRNA expression levels were calculated using the 2^−ΔΔCt method and normalized to β‐actin mRNA levels. Primer sequences used in the study were synthesized by Tsingke (Beijing, China) and were listed in Table S2.

### 2.14 Western Blot (WB) Analysis

Cells and frozen samples were lysed in RIPA extraction buffer (Beyotime, Shanghai, China, Cat# P0013B) supplemented with protease inhibitor cocktail (MedChem Express, USA, HY‐K0010), phosphatase inhibitor cocktail I (MedChem Express, USA, HY‐K0021), and phosphatase inhibitor cocktail II (MedChem Express, USA, HY‐K0022) according to the manufacturer's instructions. The lysate was then centrifuged at 14,000 × g for 15 min at 4°C to remove cellular debris. Equal amounts of protein (20 μg) were loaded into each lane, separated by 10% SDS‐PAGE, and electro‐transferred onto polyvinylidene fluoride (PVDF) membranes. The PVDF membranes were blocked with blocking buffer for 2 h at room temperature, followed by incubation with primary antibodies overnight at 4°C: mouse anti‐β‐ACTIN (Proteintech, Wuhan, China, Cat# 66009–1‐Ig, 1:1000), rabbit anti‐ANGPTL8 (Thermo Fisher Scientific, MA, USA, Cat# PA5‐38043, 1:1000), rabbit anti‐AKT (Cell Signaling Technology, MA, USA, Cat# 4691S, 1:1000), rabbit anti‐phospho‐AKT (Ser473; Cell Signaling Technology, MA, USA, Cat# 4060T, 1:1000), rabbit anti‐mTOR (Abmart, Shanghai, China, T55306, 1:1000), rabbit anti‐Phospho‐mTOR (Ser2448) (Abmart, Shanghai, China, T56571, 1:1000), rabbit anti‐S6K (Abmart, Shanghai, China, T55365, 1:1000), rabbit anti‐ Phospho‐S6K (Abmart, Shanghai, China, P90422R3, 1:1000), rabbit anti‐p16 INK4A (Santa Cruz, USA, sc‐1661, 1:1000), rabbit anti‐CDKN1Ap21 (Santa Cruz, USA, sc‐6246, 1:1000). After washing the blots three times with Tris‐buffered saline containing Tween‐20 (TBST), membranes were incubated with anti‐rabbit or mouse horseradish peroxidase (HRP)‐conjugated secondary antibody for 90 min. Protein bands were visualized and detected using an enhanced chemiluminescence system.

### 2.15 Co‐Immunoprecipitation (Co‐IP)

Cells were lysed in NP‐40 buffer supplemented with protease inhibitors and phosphatase inhibitors (MedChemExpress, NJ, USA). After centrifugation at 14,000 × g for 15 min at 4°C, protein concentrations were determined, and supernatants were collected for further analysis.

For co‐immunoprecipitation (Co‐IP) assays, both 3 T3‐L1 preadipocytes and differentiated mature adipocytes were used depending on the experimental design. 3T3‐L1 cells were maintained in Dulbecco's modified Eagle's medium (DMEM) supplemented with 10% fetal bovine serum (FBS) and induced to differentiate into mature adipocytes using a standard adipogenic cocktail (insulin, dexamethasone, and IBMX) until lipid droplet accumulation was observed, confirming successful differentiation.

For Co‐IP, 500 μg of total protein in 500 μL lysate was incubated with 5 μg of anti‐HA (Thermo Fisher Scientific, MA, USA), anti‐Flag (BioLegend, USA, #637303), or control IgG antibodies with gentle rocking at 4°C overnight, followed by incubation with Protein A/G Magnetic Beads (MedChemExpress, NJ, USA, #HY‐K0202) for 2 h at 4°C. After three washes with ice‐cold NP‐40 buffer to remove non‐specific binding, immunoprecipitated proteins were eluted and subjected to western blot analysis to assess protein–protein interactions.

### 2.16 Molecular Docking Analysis

Molecular docking analysis was performed to predict the potential interaction mode between ANGPTL8 and AKT2 proteins using HDOCKlite v1.0, a hybrid protein–protein docking platform. Prior to docking, the three‐dimensional structural models of human ANGPTL8 and AKT2 were obtained from the AlphaFold Protein Structure Database using their respective UniProt accession numbers (ANGPTL8: Q6UXH0; AKT2: P31751). The retrieved structures were preprocessed using PyMOL v2.5.3, including removal of solvent molecules and addition of polar hydrogen atoms to ensure appropriate protonation states. Protein–protein docking was conducted using the default parameters of HDOCKlite, employing a global rigid‐body docking strategy without predefined binding sites. Multiple docking poses were generated and ranked according to the HDOCK scoring function, which integrates shape complementarity and electrostatic interactions. The docking conformation with the lowest predicted docking score was selected as the most favorable complex for subsequent analysis. Visualization of the protein–protein interaction interface, as well as identification of hydrogen bonds and salt bridge interactions, was performed using PyMOL v2.5.3. The docking results were used to provide structural insights into the potential binding interface rather than to infer absolute binding affinities.

### 2.17 Enzyme‐Linked Immunosorbent Assay (ELISA)

Mouse blood samples were collected at the time of sacrifice and followed by centrifugation to obtain serum. Serum concentrations of tumor necrosis factor‐α (TNF‐α), interleukin‐6 (IL‐6), C‐X‐C motif chemokine ligand 2 (CXCL‐2), and monocyte chemoattractant protein‐1 (MCP‐1) were quantified using commercially available mouse ELISA kits (Elabscience Biotechnology Co. Ltd., Wuhan, China) in accordance with the manufacturer's instructions. All samples were assayed in duplicate. Cytokine concentrations were calculated based on standard curves generated for each analyte and are presented as ng/mL or pg/mL of serum.

### 2.18 Public Single‐Cell and Spatial Transcriptomic Data Analysis

Single‐nucleus RNA sequencing (snRNA‐seq) data of subcutaneous adipose tissue from aged and young individuals were obtained from the Gene Expression Omnibus (GEO) database (accession number: GSE235529). Additionally, Smart‐seq3xpress single‐cell transcriptomic data of young and old hepatocytes were retrieved from the ArrayExpress database (accession number: E‐MTAB‐12579), and spatial transcriptomics data of young and old liver tissues were obtained from E‐MTAB‐12809.

Raw count matrices were processed and analyzed using Scanpy (version 1.9.8) in Python. Cells with fewer than 200 detected genes or more than 10% mitochondrial gene content were excluded. Expression values were normalized using the total‐count normalization followed by logarithmic transformation. Highly variable genes were identified, and batch correction was performed with Harmony as appropriate. Dimensionality reduction was conducted by principal component analysis (PCA) followed by uniform manifold approximation and projection (UMAP).

Cell clusters were annotated based on canonical marker genes. The expression of ANGPTL8 across cell populations and spatial regions was visualized using dot plots, UMAPs, and spatial feature maps.

### 2.19 Statistical Analysis

Each experiment was independently repeated at least three times. Statistical analyzes were performed using GraphPad Prism. Data normality was assessed using the Shapiro–Wilk test prior to statistical analyzes. For comparisons between two groups, an unpaired two‐tailed Student's *t*‐test was used for normally distributed data, whereas the Mann–Whitney *U* test was applied when normality assumptions were not met. For comparisons among multiple groups, one‐way analysis of variance (ANOVA) followed by Tukey's multiple‐comparisons test was performed for normally distributed data, whereas the Kruskal–Wallis test followed by Dunn's multiple‐comparisons test was used for non‐normally distributed data. Survival curves were analyzed using the Kaplan–Meier method and compared using the log‐rank test. Data are presented as mean ± SEM unless otherwise indicated. A two‐sided *p* value < 0.05 was considered statistically significant.

## 3 Results

The Graphical Abstract provides an integrated overview of the study design and key findings, illustrating a stepwise discovery and validation framework. Briefly, large‐scale machine learning analyzes of clinical cohorts were first employed to identify ANGPTL8 as a candidate factor associated with biological aging and mortality risk. These computational findings were subsequently interrogated through in vivo and in vitro experiments to elucidate the functional and mechanistic roles of ANGPTL8 in aging‐related processes.

**FIGURE 1:**
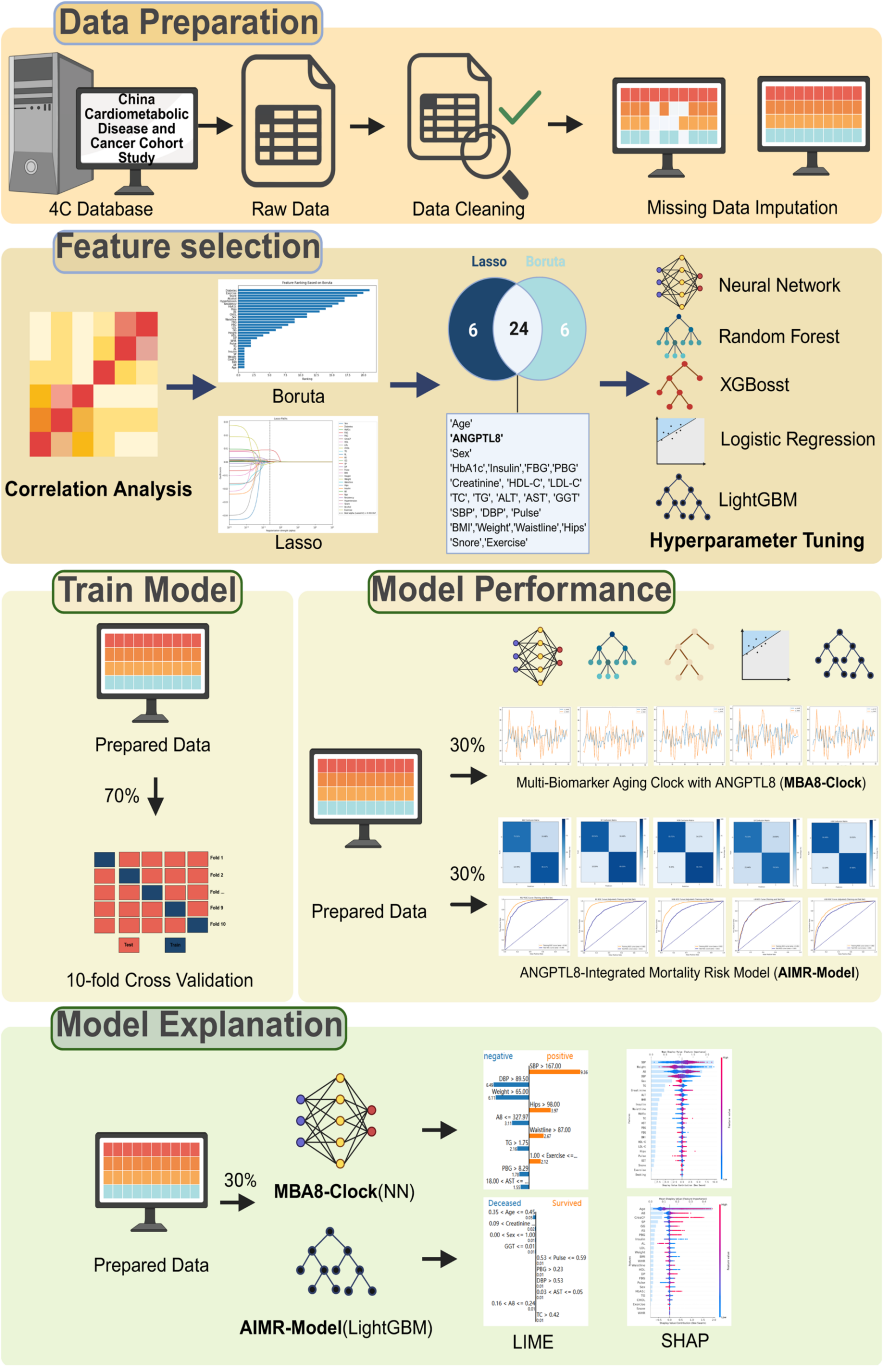
Overview of the development and analytical workflow for the MBA8‐Clock and AIMR models.

Building on this conceptual framework, Figure 1 details the analytical workflow of the machine learning component. Using data from the 4C cohort, we constructed two complementary models: the MBA8‐Clock to estimate physiological age and the AIMR‐Model to predict all‐cause mortality. Circulating ANGPTL8 levels, together with routine clinical parameters, were incorporated as input features. The workflow encompasses data preprocessing, feature selection, model training, cross‐validation, independent testing, and post hoc model interpretability analyzes using SHapley Additive exPlanations (SHAP) and Local Interpretable Model‐agnostic Explanations (LIME). The final selected variables, model coefficients, and intercepts of the optimized MBA8‐Clock and AIMR‐Model are provided in Supplementary Table S5 to facilitate transparency and independent replication. Together, these approaches established the computational foundation for identifying ANGPTL8 as a robust predictor of both biological aging and mortality.

### 3.1 Construction and Validation of the MBA8‐Clock for Biological Age Prediction

We developed and validated the MBA8‐Clock, a multi‐biomarker aging clock incorporating circulating ANGPTL8, to estimate biological age using comprehensive clinical and biochemical data from the 4C cohort. Following iterative feature ranking, 22 variables were selected, and model performance was systematically assessed across five machine learning algorithms—multilayer perceptron (MLP), random forest (RF), extreme gradient boosting (XGB), light gradient boosting machine (LGB), and logistic regression (LR)—using repeated ten‐fold cross‐validation.

**FIGURE 2:**
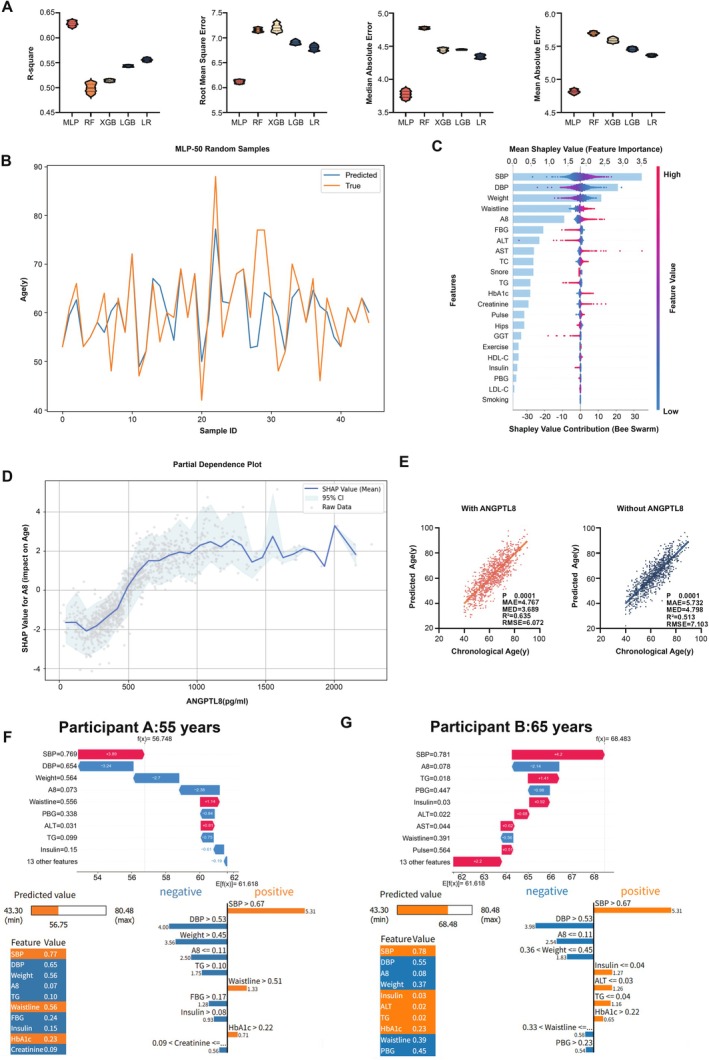
Construction, performance, and interpretability of the MBA8‐Clock. (A) Comparative performance of five machine‐learning algorithms, including multilayer perceptron (MLP), random forest (RF), extreme gradient boosting (XGB), light gradient boosting machine (LGB), and logistic regression (LR), evaluated using 100 repeated runs. Model performance was assessed by *R* ^2^, root mean square error (RMSE), median absolute error (MED), and mean absolute error (MAE). Bars represent mean values across runs, with error bars indicating standard deviation. (B) Representative comparison between predicted biological age and chronological age for 50 randomly selected participants using the MLP‐based MBA8‐Clock. Lines depict individual trajectories, illustrating the concordance between predicted and actual age estimates. (C) SHapley Additive exPlanations (SHAP) beeswarm plot summarizing the global feature importance of all predictors included in the MBA8‐Clock. Features are ranked by mean absolute SHAP value. Each point represents one individual, with color indicating the relative feature value (low to high). (D) Partial dependence plot illustrating the nonlinear relationship between circulating ANGPTL8 levels and predicted biological age in the MBA8‐Clock. Shaded areas denote 95% confidence intervals. (E) Scatter plots comparing predicted biological age versus chronological age in models trained with (left) or without (right) ANGPTL8. Removal of ANGPTL8 resulted in visibly reduced concordance and impaired predictive performance. (F) Individual‐level model interpretation for a representative 55‐year‐old participant using SHAP (up) and LIME (down) analyzes, illustrating feature contributions to the predicted biological age. (G) Individual‐level SHAP and LIME explanations for a representative 65‐year‐old participant, demonstrating consistent feature attribution patterns across different ages and supporting the robustness of model interpretability.

Among all evaluated models, the MLP algorithm consistently demonstrated superior predictive performance. As shown in Figure 2A, the MLP‐based MBA8‐Clock achieved a mean coefficient of determination (*R* ^2^) of 0.635, a median absolute deviation error (MED) of 3.689 years, a mean absolute error (MAE) of 4.767 years, and a root mean square error (RMSE) of 6.072 ± 0.03 years. Given the chronological age range of 23–98 years in the cohort, this corresponds to a relative prediction error of approximately 8.1%, indicating high precision in biological age estimation.

Visualization of predicted versus chronological age revealed strong concordance between model outputs and actual age (Figure 2B), supporting the robustness and stability of the MBA8‐Clock. To interrogate model interpretability, post hoc analyzes using SHAP were applied to the MLP model, enabling both global and individual‐level dissection of feature contributions. SHAP summary analysis (Figure 2C) ranked predictors according to their mean absolute SHAP values in the internal validation cohort, thereby quantifying the relative contribution of each feature to biological age prediction. Positive SHAP values indicate factors associated with increased predicted biological age, whereas negative values reflect relative protective associations.

Among the top‐ranked predictors, systolic blood pressure (SBP), waist circumference, and circulating ANGPTL8 levels exhibited predominantly positive SHAP values, suggesting that increased vascular load, central adiposity, and ANGPTL8‐related metabolic signaling contribute to accelerated physiological aging. In contrast, body weight and diastolic blood pressure (DBP) showed largely negative SHAP values, indicating inverse associations with predicted biological age. These SHAP‐derived patterns closely align with well‐established hallmarks of aging, including vascular stiffening, altered body composition, and metabolic dysregulation. Notably, the prominence of ANGPTL8 alongside classical clinical parameters supports its relevance as a systemic aging‐associated factor rather than a model‐specific artifact.

Consistent with the SHAP summary results, circulating ANGPTL8 levels demonstrated a uniformly positive contribution to predicted biological age across individuals. Partial dependence analysis (Figure 2D) further revealed a nonlinear association between ANGPTL8 and biological age, with a pronounced increase in its effect at concentrations exceeding approximately 250 pg/mL. Importantly, this relationship persisted after adjustment for other age‐related clinical variables included in the model, indicating that ANGPTL8 provides information beyond conventional risk factors. To evaluate whether ANGPTL8 contributes non‐redundant predictive value, the MLP model was retrained under identical conditions following exclusion of ANGPTL8. As shown in Figure 2E, removal of ANGPTL8 resulted in consistent deterioration of model performance across multiple metrics, including MAE, MED, *R* ^2^, and RMSE. These findings indicate that ANGPTL8 substantially enhances the predictive capacity of the MBA8‐Clock and represents an integral determinant of biological age estimation.

To further elucidate individual‐level decision mechanisms and the personalized contribution of ANGPTL8, case‐based interpretability analyzes were conducted using SHAP and LIME. Two representative participants were randomly selected to illustrate how individual clinical features jointly shaped predicted biological age (Figure 2F,G; detailed characteristics in Table S3). Participant A, a 55‐year‐old individual with markedly elevated circulating ANGPTL8 levels (819.12 pg/mL), exhibited a predicted biological age of 56.75 years. Both SHAP and LIME analyzes indicated that elevated ANGPTL8, together with increased systolic blood pressure and HbA1c, contributed positively to age acceleration. Although several features exerted modest protective effects, the combined influence of ANGPTL8 and vascular–metabolic risk factors dominated the model output. Participant B was a 65‐year‐old male with relatively low circulating ANGPTL8 levels (198 pg/mL) and a predicted biological age of 68.48 years. Despite the negative contribution of lower ANGPTL8 levels, elevated systolic blood pressure exerted a dominant positive effect, outweighing the protective influence of ANGPTL8 and resulting in an older biological age estimate. Notably, ANGPTL8 exhibited a consistent directional effect across both individuals, with its impact modulated by the broader cardiometabolic context. Collectively, these population‐level and individual‐level analyzes demonstrate that the MBA8‐Clock robustly captures biological aging and that circulating ANGPTL8 represents a consistent, biologically meaningful contributor to age prediction across individuals.

### 3.2 Development and Performance Evaluation of the AIMR‐Model for Mortality Prediction

Using the same cohort and modeling framework described above, we developed a 10‐year all‐cause mortality risk prediction model. To reduce multicollinearity, variables with Pearson correlation coefficients greater than 0.85 were excluded prior to model construction. Feature selection was subsequently performed using a combination of LASSO and Boruta algorithms, while circulating ANGPTL8 concentration was retained for evaluation of its independent contribution. Five machine learning algorithms—RF, XGB, LGB, LR, and MLP—were implemented and systematically compared.

**FIGURE 3:**
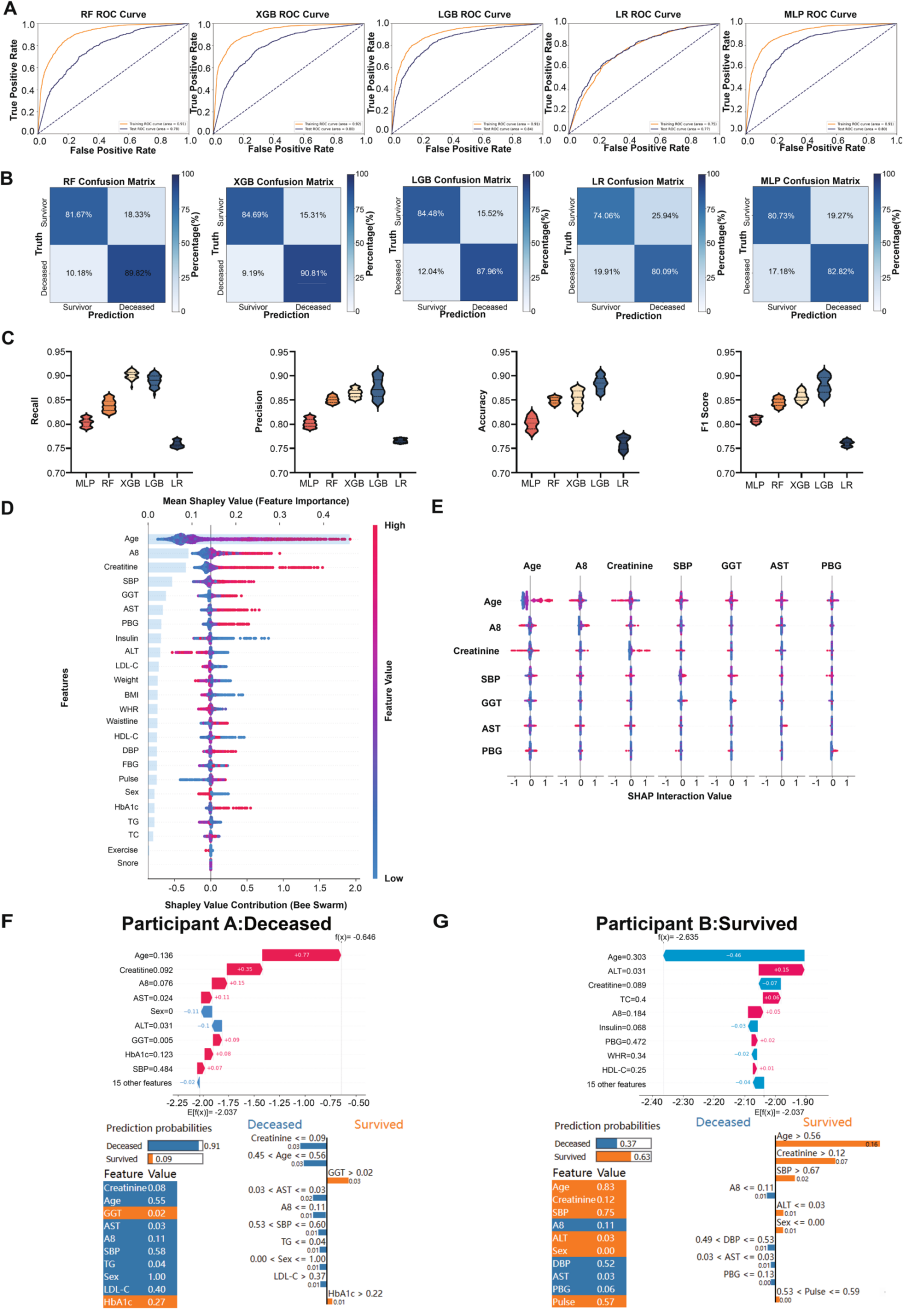
Development, performance evaluation, and interpretability of the AIMR‐Model. (A) Receiver operating characteristic (ROC) curves of five machine learning algorithms, including random forest (RF), extreme gradient boosting (XGB), light gradient boosting machine (LGB), logistic regression (LR), and multilayer perceptron (MLP), for mortality risk prediction. (B) Confusion matrices of RF, XGB, LGB, LR, and MLP models illustrating classification performance in the test set. (C) Bar plots summarizing recall, precision, accuracy, and F1 score for each model, based on 100 repeated runs to assess model stability and robustness. (D) SHapley Additive exPlanations (SHAP) beeswarm plot of the final MBA8‐Clock, displaying the global importance and directional effects of individual features on model output. (E) SHAP interaction value analysis of the top seven contributing features, including Age, ANGPTL8 (A8), creatinine, systolic blood pressure (SBP), γ‐glutamyl transferase (GGT), aspartate aminotransferase (AST), and postprandial blood glucose (PBG), highlighting pairwise feature interactions within the model. (F) Representative SHAP and LIME explanations for a randomly selected participant who died during the 10‐year follow‐up period, illustrating individualized risk attribution. (G) Representative SHAP and LIME explanations for a randomly selected participant who survived throughout the 10‐year follow‐up period, demonstrating feature contributions at the individual level.

Model performance was evaluated using 100 iterations of stratified ten‐fold cross‐validation and assessed by multiple complementary metrics, including area under the receiver operating characteristic curve (AUC), accuracy, sensitivity, specificity, precision, F1 score, and log loss in both training and validation datasets. As shown in Figure 3A, all five models demonstrated robust discriminative ability for 10‐year mortality prediction, with validation‐set AUCs of 0.78 (RF), 0.80 (XGB), 0.84 (LGB), 0.77 (LR), and 0.80 (MLP). Gradient boosting–based approaches, particularly LGB and XGB, achieved the highest AUC values, indicating superior discrimination between survivors and non‐survivors.

Consistent with these findings, classification performance analyzes revealed well‐balanced confusion matrices across all models, suggesting stable separation of survival outcomes over repeated training iterations. Notably, both XGB and LGB achieved classification accuracies exceeding 85%, with consistently high sensitivity and no evidence of overfitting (Figure 3B). Among all evaluated algorithms, XGB demonstrated the most favorable overall performance. Across 100 repeated stratified cross‐validation runs, the XGB‐based model achieved an average recall of 0.90 ± 0.01, precision of 0.86 ± 0.01, accuracy of 0.85 ± 0.02, and an F1 score of 0.86 ± 0.01, accompanied by a low log loss of 0.18 ± 0.002 (Figure 3C). Given its balanced performance across discrimination, calibration, and stability metrics, XGB was selected as the final algorithm for the AIMR‐Model.

To elucidate the key determinants underlying mortality prediction, SHAP analysis was applied to the XGB‐based AIMR‐Model. As shown in Figure 3D, chronological age emerged as the strongest contributor to mortality risk, followed by circulating ANGPTL8 concentration. In addition to age and ANGPTL8, several established clinical parameters—including circulating creatinine, systolic blood pressure (SBP), and gamma‐glutamyl transferase (GGT)—exhibited positive SHAP values, indicating higher predicted mortality risk with increasing levels. Collectively, these results highlight a convergence of aging, renal dysfunction, hepatic impairment, vascular stress, and ANGPTL8‐associated metabolic signaling as major contributors to long‐term mortality risk.

Consistent with our previous observations linking elevated ANGPTL8 levels to age‐associated conditions such as non‐alcoholic fatty liver disease, diabetic cognitive impairment, and diabetic nephropathy, analysis of the 4C cohort demonstrated enrichment of circulating ANGPTL8 among individuals with aging‐related morbidities, further supporting its relevance across diverse age‐related disease contexts. SHAP interaction analysis (Figure 3E) further revealed synergistic effects between age and multiple high‐impact features, including ANGPTL8, creatinine, GGT, and SBP. Notably, the combination of advanced age and elevated ANGPTL8 consistently shifted model predictions toward higher mortality risk, suggesting that ANGPTL8 may amplify age‐related clinical vulnerability rather than acting solely as an isolated risk marker.

To illustrate individual‐level interpretability, two representative participants were randomly selected for case‐based analysis. Individual clinical characteristics are summarized in Table S4. SHAP and LIME analyzes were applied to delineate the contribution of specific features to each participant's predicted 10‐year mortality risk.

As shown in Figure 3F, Participant A died during the 10‐year follow‐up period, and the AIMR‐Model estimated a high mortality probability of 91%. Interpretability analyzes consistently identified advanced age, impaired renal function (elevated creatinine), abnormal liver enzymes (AST and GGT), and elevated circulating ANGPTL8 as dominant contributors driving the high‐risk prediction, in close concordance with the observed outcome. In contrast, Participant B survived throughout the follow‐up period, for whom the AIMR‐Model predicted a substantially lower mortality probability of 37%. Although moderately elevated ANGPTL8 exerted a modest positive contribution to predicted risk, younger age and preserved hepatic and renal function acted as dominant protective factors, collectively shifting the model toward a favorable survival prediction.

Together, these population‐level and individual‐level analyzes demonstrate that the AIMR‐Model achieves robust predictive performance while providing biologically coherent and clinically interpretable explanations, supporting its utility for personalized mortality risk stratification.

### 3.3 ANGPTL8 as a Key Cross‐Link Between Physiological Aging and Mortality Risk

Building on the individual‐level interpretability analyzes, we next sought to define population‐level clinical and biological features that distinguish divergent trajectories of biological aging and long‐term mortality risk.

To characterize phenotypic correlates of biological aging, participants were stratified according to MBA8‐Clock prediction deviation into an older‐predicted group (biological age exceeding chronological age by > 5 years) and a younger‐predicted group (biological age lower than chronological age by > 5 years). SHAP‐based subgroup analysis identified five dominant contributors to biological age prediction: circulating ANGPTL8, systolic blood pressure (SBP), diastolic blood pressure (DBP), serum creatinine, and body weight.

**FIGURE 4:**
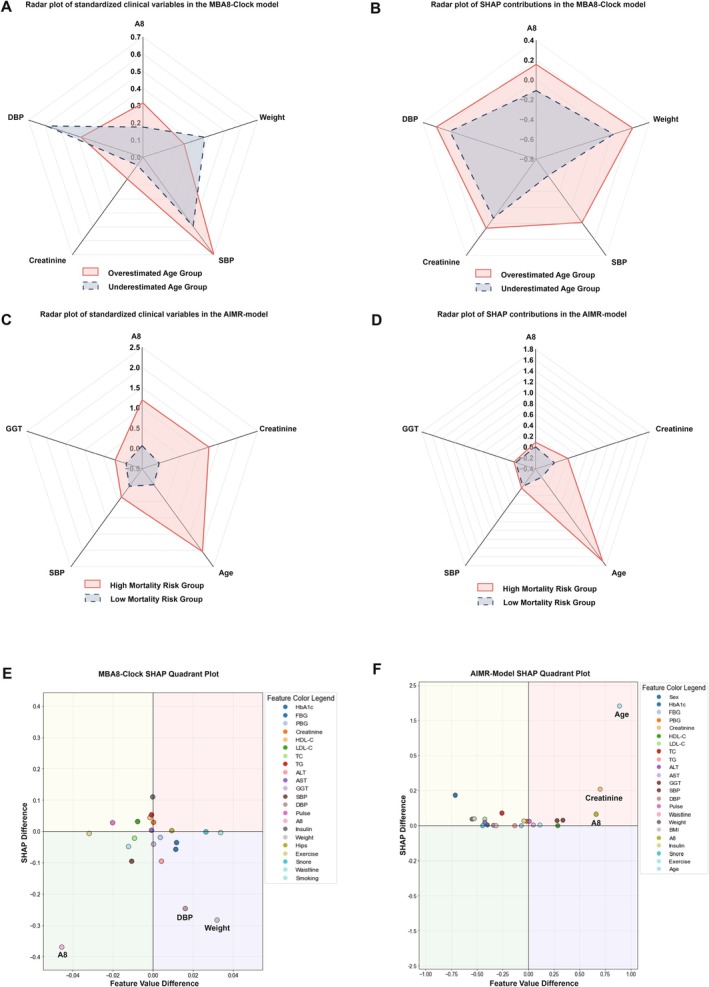
Phenotypic stratification and SHAP‐based characterization of subgroups defined by the MBA8‐Clock and AIMR‐Model. (A) Radar plot comparing standardized clinical values of the top five SHAP‐ranked features, including ANGPTL8, diastolic blood pressure (DBP), creatinine, systolic blood pressure (SBP), and body weight, between two subgroups stratified by the MBA8‐Clock (individuals with age overestimation versus age underestimation). The radar plot illustrates markedly distinct phenotypic profiles between the two subgroups. (B) Radar plot integrating SHAP values of the same two MBA8‐Clock‐defined subgroups, highlighting differences in feature contributions to biological age acceleration or deceleration. (C) Radar plot comparing standardized clinical values of the top five SHAP‐ranked features, including ANGPTL8, γ‐glutamyl transferase (GGT), SBP, age, and creatinine, between two subgroups stratified by the AIMR‐Model (individuals with high predicted mortality risk versus low predicted mortality risk). Distinct phenotypic patterns are observed between the two mortality risk strata. (D) Radar plot integrating SHAP values of the AIMR‐Model‐defined high‐ and low‐risk subgroups, demonstrating differential feature contributions to mortality risk prediction. (E) Four‐quadrant plot of SHAP values comparing the two MBA8‐Clock‐defined subgroups, enabling visualization of concordant and discordant feature effects between the populations. (F) Four‐quadrant plot of SHAP values comparing the high‐ and low‐risk mortality subgroups, illustrating subgroup‐specific feature importance and directional effects within the AIMR‐Model.

Radar plot visualization of standardized clinical variables revealed clearly divergent phenotypic profiles between the two subgroups (Figure 4A). Compared with the older‐predicted group, individuals in the younger‐predicted group exhibited lower circulating ANGPTL8 and creatinine levels, lower SBP with relatively higher DBP, and a tendency toward higher body weight. These coordinated features collectively reflect preserved metabolic, renal, and vascular function, as well as reduced frailty burden.

Consistent with these phenotypic patterns, all five variables displayed positive SHAP contributions in the older‐predicted group (Figure 4B), indicating that higher ANGPTL8, elevated creatinine, increased SBP, reduced DBP, and lower body weight jointly shifted the MBA8‐Clock toward older biological age estimates.

To integrate subgroup‐level phenotypic differences with their functional impact on age prediction, we constructed a quadrant‐based SHAP interaction framework (Figure 4C). Features located in the upper‐right quadrant exhibited both higher clinical values and stronger positive SHAP effects in the older‐predicted group, thereby acting as pro‐aging drivers within the model. Serum creatinine prominently occupied this quadrant, indicating that renal dysfunction both manifests phenotypically and exerts a strong upward influence on biological age estimation. In contrast, ANGPTL8 and SBP localized to the lower‐left quadrant, reflecting lower levels and age‐lowering SHAP contributions in the younger‐predicted subgroup. This two‐dimensional framework provides an integrated view linking clinical variation to functional aging effects.

An analogous stratification strategy was applied for mortality risk prediction. Participants were classified into a low‐risk group and a high‐risk group based on the predicted mortality probability generated by the AIMR model, with a cutoff of 30% (predicted mortality risk < 30% vs. ≥ 30%). SHAP analysis identified circulating ANGPTL8, chronological age, serum creatinine, gamma‐glutamyl transferase (GGT), and SBP as the principal contributors to 10‐year mortality risk. As shown in Figure 4D, individuals in the high‐risk subgroup exhibited markedly higher ANGPTL8 levels, older age, elevated creatinine and GGT concentrations, and increased SBP compared with those in the low‐risk subgroup. These features reflect convergent metabolic stress, renal and hepatic dysfunction, and vascular burden, collectively shifting model predictions toward higher mortality risk. Consistently, SHAP radar analysis demonstrated close alignment between subgroup‐level clinical differences and their directional contributions to mortality prediction (Figure 4E), with chronological age exerting the strongest overall effect. The quadrant‐based SHAP interaction plot (Figure 4F) further integrated phenotypic differences with functional contributions to mortality risk. Chronological age occupied the dominant upper‐right quadrant, followed by circulating ANGPTL8 and serum creatinine, indicating that elevations in these variables consistently coincided with strong positive SHAP effects driving higher predicted mortality risk.

To further evaluate the clinical relevance of ANGPTL8 in human aging, participants were stratified into quartiles according to circulating ANGPTL8 concentrations. Frailty index scores increased progressively across ANGPTL8 quartiles, indicating a significant association between elevated ANGPTL8 levels and age‐related functional impairment (Figure S2A). Similarly, the metabolic score for visceral fat (METS‐VF) values showed a stepwise increase with rising ANGPTL8 concentrations, suggesting a close relationship between ANGPTL8 and adverse visceral metabolic remodeling (Figure S2B). These findings further support the association between circulating ANGPTL8 and clinically relevant aging phenotypes in humans.

Collectively, these analyzes identify a shared constellation of aging‐associated clinical features—including blood pressure indices, renal and hepatic function markers, and metabolic stress indicators—that coherently shape both biological age estimation and long‐term mortality risk. Importantly, ANGPTL8 emerged as a recurrent contributor across both predictive frameworks, with elevated levels aligning with accelerated biological aging and increased mortality risk. This convergence supports a model in which ANGPTL8 represents a biological link connecting physiological aging with adverse survival outcomes, thereby providing a strong rationale for the mechanistic investigations presented in the subsequent sections.

### 3.4 Loss of *Angptl8* Mitigates Age‐Associated Phenotypes in Mice

Building on our MBA8‐Clock and AIMR‐Model analyzes derived from large‐scale human clinical datasets, which consistently identified circulating ANGPTL8 as a robust correlate of biological aging and mortality risk, importantly, these population‐based machine learning analyzes identify ANGPTL8 as an aging‐associated predictor but do not by themselves establish a causal relationship. Therefore, we subsequently performed genetic and mechanistic studies in mice to determine whether ANGPTL8 directly contributes to aging‐related phenotypes.

**FIGURE 5:**
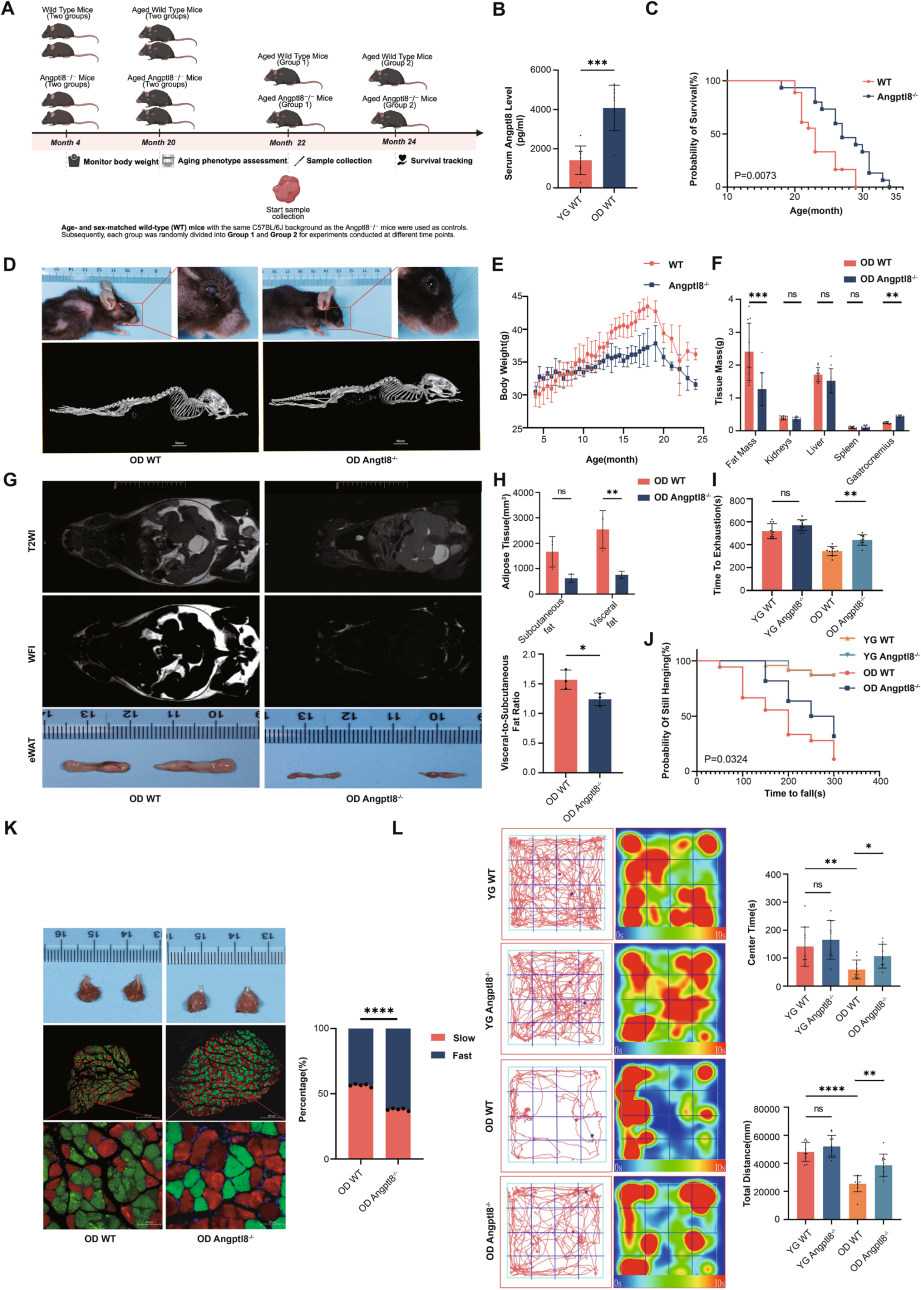
Genetic ablation of *Angptl8* attenuates aging‐associated phenotypes, preserves musculoskeletal integrity, and improves functional performance in mice. (A) Schematic overview of the experimental design. Homozygous *Angptl8* knockout (*Angptl8* ^*−/−*^) mice were generated on a C57BL/6J background, with age‐ and sex‐matched wild‐type (WT) littermates used as controls. Mice of both genotypes were enrolled at 4 months of age and randomly assigned to two predefined cohorts. One cohort was sacrificed at 22 months of age for comprehensive tissue‐level, histological, and molecular analyzes, whereas the second cohort was longitudinally followed until natural death to assess lifespan and survival outcomes. (B) Bar graph comparing circulating ANGPTL8 protein concentrations in serum from young (Young, *n* = 9) and aged (Old, *n* = 9) mice, demonstrating an age‐associated increase in ANGPTL8 levels. Data are presented as mean ± SEM. Statistical significance was determined using an unpaired two‐tailed Student's *t*‐test. *p < 0.05, **p < 0.01, ***p < 0.001. (C) Kaplan–Meier survival curves comparing overall survival between WT mice (*n* = 10) and *Angptl8* ^*−/−*^ mice (*n* = 10). Mice were monitored until spontaneous death or predefined humane endpoints according to institutional animal welfare guidelines. Animals reaching humane endpoints were recorded as survival events. All deceased animals underwent postmortem examination to exclude non‐aging‐related causes of death. Survival differences were analyzed using the log‐rank (Mantel–Cox) test. (D) Representative gross appearance of aged WT and age‐matched *Angptl8* ^*−/−*^ mice at 24 months of age. Enlarged views of the eye region (right panels) highlight cataract formation. Corresponding micro‐computed tomography (micro‐CT) images (lower panels) from the same mice illustrate age‐associated spinal curvature. Scale bar, 10 mm. (E) Longitudinal body weight trajectories of WT mice (*n* = 10) and *Angptl8* ^*−/−*^ mice (*n* = 10) recorded from 4 to 24 months of age. (F) Bar graphs showing weights of selected organs in 22‐month‐old wild‐type (WT) mice (*n* = 10) and age‐matched *Angptl8* ^*−/−*^ mice (*n* = 10). Organs analyzed included adipose tissue (combined epididymal and inguinal fat pads), kidney, liver, spleen, and skeletal muscle. Statistical comparisons between groups were performed using unpaired two‐tailed Student's *t*‐test for each organ. Data are presented as mean ± SEM. ns, *p* > 0.05, *p < 0.05, **p < 0.01, ***p < 0.001. (G) Representative transverse T2‐weighted MRI images (top) and whole‐body fat imaging (middle) of 22‐month‐old wild‐type (WT) and *Angptl8* ^*−/−*^ mice. Gross images of epididymal white adipose tissue (bottom) were obtained at dissection to validate MRI‐based observations. WT mice are shown on the left (OD WT), and *Angptl8* ^*−/−*^ mice are shown on the right (OD *Angptl8* ^*−/−*^). Scale bar, 30 mm. (H) Quantification of total adipose tissue volume (mm^3^) (upper panel) and visceral‐to‐subcutaneous fat ratio (lower panel) in 22‐month‐old wild‐type (WT) and *Angptl8* ^*−/−*^ mice (*n* = 3 per group). Adipose tissue volumes were derived from MRI‐based volumetric reconstruction. Statistical comparisons between groups were performed using the Mann–Whitney *U* test due to non‐normal distribution and small sample size. Data are presented as mean ± SEM. ns, *p* > 0.05; *p < 0.05. (I) Treadmill endurance test showing time to exhaustion in 20‐month‐old wild‐type (WT) mice (*n* = 10) and age‐matched *Angptl8* ^*−/−*^ mice (*n* = 10). Statistical comparisons between groups were performed using an unpaired two‐tailed Student's *t*‐test. Data are presented as mean ± SEM. ns, *p* > 0.05; *p < 0.05; **p < 0.01. (J) Rotarod performance of 20‐month‐old wild‐type (WT) and *Angptl8* ^*−/−*^ mice, as well as 4‐month‐old young (YG) WT and YG *Angptl8* ^*−/−*^ mice (*n* = 10 per group). Mice were placed on an accelerating rotarod starting from rest, with a maximum test duration of 300 s. Rotarod performance was analyzed using Kaplan–Meier survival curves based on latency to fall, and group differences were assessed using the log‐rank (Mantel–Cox) test. Statistical analysis showed no significant difference between YG WT and YG *Angptl8* ^*−/−*^ mice (*p* > 0.05), whereas aged (20‐month‐old) *Angptl8* ^*−/−*^ mice exhibited a significantly prolonged latency to fall compared with age‐matched WT controls (*p* = 0.0324). Data are presented as survival curves representing time to fall. (K) Representative morphology and fiber‐type composition of the soleus muscle in aged mice. Top: Gross morphology of the soleus muscle harvested from 22‐month‐old WT and *Angptl8* ^*−/−*^ mice at sacrifice. Middle: Representative immunofluorescence staining of soleus cross‐sections from WT (*n* = 5) and age‐matched *Angptl8* ^*−/−*^ mice (*n* = 5). Fast‐twitch myofibers are labeled with Cy3 (red; anti‐fast myosin skeletal), and slow‐twitch myofibers are labeled with Alexa Fluor 488 (green; anti‐slow myosin skeletal). Scale bar, 500 μm. (L) Bottom: Magnified view of the stained sections to illustrate muscle fiber morphology and type composition. Scale bar, 50 μm. Open‐field test assessing spontaneous locomotor activity in 20‐month‐old WT and *Angptl8* ^*−/−*^ mice, as well as 4‐month‐old young (YG) WT and YG *Angptl8* ^*−/−*^ mice (*n* = 10 per group). Representative movement trajectories and positional heatmaps are shown. Quantitative analysis of total travel distance and time spent in the central zone is presented as bar graphs. Statistical comparisons among groups were performed using one‐way ANOVA followed by Tukey's multiple comparisons test. Data are presented as mean ± SEM. ns, *p* > 0.05, *p < 0.05, **p < 0.01, ***p < 0.001, ****p < 0.0001.

To investigate the role of ANGPTL8 in aging, we generated homozygous *Angptl8* knockout (*Angptl8* ^*−/−*^) mice on a C57BL/6J background and used age‐ and sex‐matched wild‐type (WT) littermates as controls. As illustrated in Figure 5A, mice were enrolled at 4 months of age and randomly assigned to one of two predefined cohorts. One cohort was sacrificed at 22 months for tissue collection and molecular analyzes, whereas the second cohort was monitored longitudinally until natural death for survival assessment. We next quantified circulating ANGPTL8 levels in young (4‐month‐old) and aged (22‐month‐old) C57BL/6J mice. Consistent with our observations in the human cohort, aged mice exhibited significantly higher serum ANGPTL8 concentrations than young controls (Figure 5B), indicating that age‐associated upregulation of ANGPTL8 is evolutionarily conserved across species. These findings, together with our population‐based analyzes, suggest a potential role for ANGPTL8 in the regulation of aging‐related processes and provide a rationale for investigating the impact of *Angptl8* deficiency on organismal aging.

Kaplan–Meier survival analysis revealed a clear divergence in survival probability between genotypes during late life, with WT mice exhibiting accelerated mortality beginning in advanced age. In contrast, *Angptl8* ^*−/−*^ mice displayed significantly improved survival and an extended lifespan relative to WT controls (Figure 5C), indicating that loss of *Angptl8* confers a survival advantage during aging. Consistent with improved survival, aged *Angptl8* ^*−/−*^ mice exhibited attenuation of multiple overt aging‐associated physical phenotypes. At 24 months of age, WT mice displayed characteristic signs of advanced aging, including fur graying and loss, alopecia, cataract formation, and spinal kyphosis, as assessed by gross inspection, ocular examination, and CT‐based spinal imaging. These features were substantially alleviated in age‐matched *Angptl8* ^*−/−*^ mice (Figure 5D), indicating delayed manifestation of visible aging traits.

To further characterize systemic aging trajectories, body weight was monitored longitudinally from 4 to 24 months of age. Both genotypes exhibited comparable growth patterns during early adulthood, with no significant differences observed before 10 months of age. However, from midlife onward, *Angptl8* ^*−/−*^ mice consistently maintained lower body weight compared with WT controls (Figure 5E), indicating a divergence in age‐associated weight trajectories. To identify the tissue sources underlying this difference, we next quantified the weights of major organs and tissues in aged mice. This analysis revealed that the body weight reduction in *Angptl8* ^*−/−*^ mice was primarily attributable to marked differences in adipose tissue and skeletal muscle mass (Figure 5F). The total adipose tissue mass was significantly reduced in *Angptl8* ^*−/−*^ mice compared to WT controls. In contrast, the skeletal muscle mass, as indicated by the soleus muscle, was largely preserved in *Angptl8* ^*−/−*^ mice. These findings suggest that the deficiency of *Angptl8* reduces the accumulation of adipose tissue while maintaining skeletal muscle mass during aging.

Given that aging is typically characterized by an increase in visceral adiposity accompanied by a relative decline in subcutaneous fat (Muzumdar et al. 2008), we next investigated whether *Angptl8* deficiency influences systemic adipose tissue distribution in aged mice. To this end, whole‐body magnetic resonance imaging (MRI) was performed in 22‐month‐old wild‐type (WT) and *Angptl8* ^*−/−*^ mice, followed by quantitative analysis of visceral and subcutaneous fat volumes. MRI‐based volumetric analysis revealed that *Angptl8* deficiency significantly reduced both visceral and subcutaneous adipose tissue volumes in aged mice (Figure 5G). Notably, the reduction in visceral fat was more pronounced than that observed in subcutaneous fat. Consistently, analysis of the visceral‐to‐subcutaneous fat ratio demonstrated that although both WT and *Angptl8* ^*−/−*^ mice exhibited a ratio greater than one (Yamashita et al. 1996), indicative of an age‐associated visceral fat–dominant distribution, *Angptl8* deletion markedly attenuated visceral fat accumulation relative to subcutaneous fat (Figures 5H and S3A,B). These findings indicate that *Angptl8* deficiency not only reduces total adiposity during aging but also partially remodels the age‐associated fat distribution pattern by preferentially limiting visceral fat expansion.

To determine whether the preserved muscle mass observed in aged *Angptl8* ^*−/−*^ mice translated into functional benefits, we first assessed whole‐body exercise capacity using the treadmill exhaustion test. Aged *Angptl8* ^*−/−*^ mice exhibited a significantly prolonged time to exhaustion compared with age‐matched WT controls (Figure 5I), indicating enhanced endurance capacity and resistance to age‐associated functional decline. We next evaluated neuromuscular coordination and motor performance using the accelerating rotarod assay. Consistently, *Angptl8* ^*−/−*^ mice showed a significantly increased latency to fall compared with WT mice (Figure 5J), suggesting improved balance and motor coordination in aged animals.

To further characterize the structural basis underlying these functional improvements, we examined the soleus muscle, a postural muscle highly susceptible to age‐related degeneration (Tieland et al. 2018). Gross examination revealed that the soleus muscle from aged *Angptl8* ^*−/−*^ mice appeared visibly larger and more robust than that from WT controls (Figure 5K). Consistent with these macroscopic observations, histological analysis demonstrated significantly increased muscle fiber thickness in *Angptl8* ^*−/−*^ mice compared with WT controls. In addition, muscle fiber–type staining indicated a higher proportion of fast‐twitch fibers in *Angptl8* ^*−/−*^ mice. Collectively, these findings suggest preserved muscle structural integrity and a shift toward a more youthful muscle phenotype in the absence of *Angptl8*.

We further evaluated spontaneous locomotor activity and exploratory behavior using the open field test (OFT). As expected, aging was associated with a general reduction in locomotion in both genotypes. Notably, however, aged *Angptl8* ^*−/−*^ mice traveled significantly greater total distances and spent more time in the central zone compared with WT mice (Figure 5L), suggesting attenuation of age‐related declines in physical activity and exploratory behavior.

Collectively, these results demonstrate that genetic ablation of *Angptl8* not only prolongs lifespan but also broadly mitigates multiple hallmarks of systemic aging, including adipose expansion, muscle loss, neuromuscular deterioration, and behavioral decline. These findings establish *Angptl8* as a functional regulator of age‐associated physiological deterioration and provide in vivo causal evidence linking ANGPTL8 signaling to organismal aging.

### 3.5 ANGPTL8 Promotes Adipocyte Senescence Through a Cell‐Intrinsic Mechanism

**FIGURE 6:**
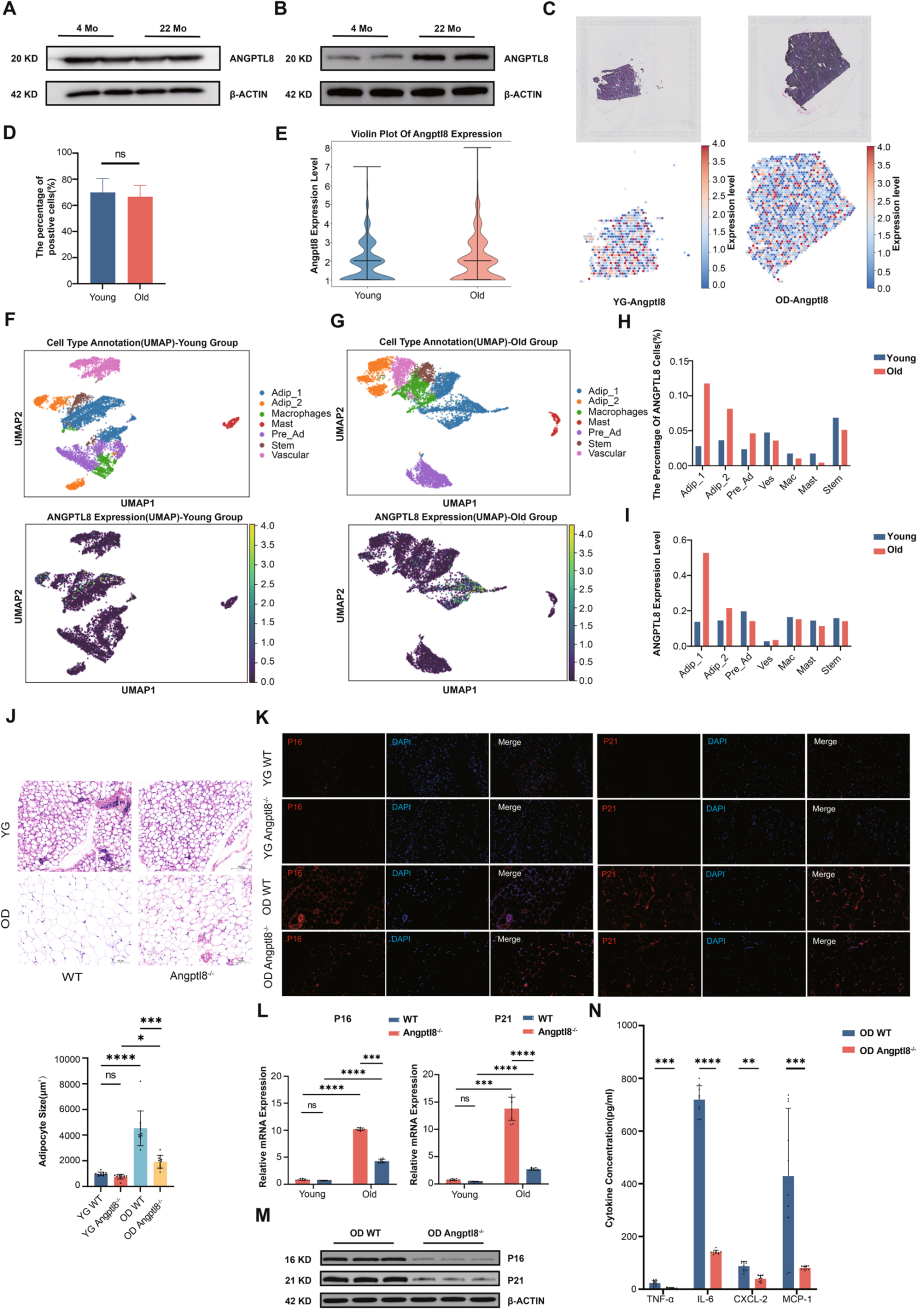
Age‐associated upregulation and adipose‐enriched expression of Angptl8 drive adipocyte senescence in mice and humans. (A) Representative western blot analysis of ANGPTL8 protein expression in liver tissues from young (4‐month‐old) and aged (22‐month‐old) mice. (B) Representative western blot analysis of ANGPTL8 protein expression in epididymal white adipose tissues from young (4‐month‐old) and aged (22‐month‐old) mice. (C) Representative liver tissue sections from young (left) and aged (right) mice, with corresponding spatial localization of ANGPTL8 protein, illustrating age‐dependent changes in its distribution. (D) Quantification of the proportion of hepatocytes expressing *Angptl8* in liver tissues from young and aged mice. (E) Quantitative analysis of *Angptl8* expression intensity in hepatocytes from young and aged mouse livers. UMAP visualization of single‐cell transcriptomic profiles from abdominal adipose tissue of young human donors. Distinct colors denote annotated cell clusters (legend on the right). Lower panels summarize the proportion of *ANGPTL8*‐expressing cells and the corresponding expression intensity across clusters. (F, G) UMAP visualization of single‐cell transcriptomic profiles from abdominal adipose tissue of elderly human donors. Distinct colors denote annotated cell clusters (legend on the right). Lower panels summarize the proportion of *ANGPTL8*‐expressing cells and the corresponding expression intensity across clusters. (H) Bar graph summarizing the proportion of *ANGPTL8*‐positive cells across individual cell clusters in human abdominal adipose tissue. (I) Bar graph summarizing *ANGPTL8* expression intensity across individual cell clusters in human abdominal adipose tissue. (J) Hematoxylin and eosin (H&E) staining of visceral adipose tissue from young (4‐month‐old) and aged (22‐month‐old) WT and *Angptl8* ^*−/−*^ mice. Scale bar, 100 μm. Lower panels show quantitative analysis of adipocyte cell size. (K) Representative immunofluorescence staining of visceral adipose tissue from young (4‐month‐old) and aged (22‐month‐old) WT and *Angptl8* ^*−/−*^ mice, showing p16 (red), p21 (brown‐red), and nuclear counterstaining with DAPI (blue). Scale bar, 50 μm. (L) Quantitative real‐time PCR analysis of p16 and p21 mRNA expression levels in visceral adipose tissue from young wild‐type (YG WT, 4‐month‐old; *n* = 10), old wild‐type (OD WT, 22‐month‐old; *n* = 10), young *Angptl8* ^*−/−*^ (YG KO, 4‐month‐old; *n* = 10) and old *Angptl8* ^*−/−*^ (OD KO, 22‐month‐old; *n* = 10) mice, with β‐Actin used as the internal control. Data are presented as mean ± SEM. Normality was assessed using the Shapiro–Wilk test. Statistical significance was determined by one‐way ANOVA followed by Tukey's multiple‐comparisons test. ns, *p* > 0.05; *p < 0.05; **p < 0.01; ***p < 0.001; ****p < 0.0001. (M) Representative western blot analysis of p16 and p21 protein expression in visceral adipose tissue from young (4‐month‐old) and aged (22‐month‐old) WT and *Angptl8* ^*−/−*^ mice. (N) Circulating and adipose‐associated senescence‐associated secretory phenotype (SASP) factor concentrations measured in visceral adipose tissue from young (4‐month‐old) and aged (22‐month‐old) WT mice (*n* = 10) and Angptl8^−^/^−^ mice (*n* = 10), linking *Angptl8*‐driven adipocyte senescence to systemic inflammatory output.

Given that circulating ANGPTL8 levels progressively increase with chronological age in both humans and mice, and that genetic ablation of *Angptl8* markedly attenuates systemic aging phenotypes, we next sought to identify the principal tissue source underlying this age‐associated elevation and to determine its contribution to adipose tissue aging. ANGPTL8 is predominantly expressed and secreted by the liver and adipose tissue; therefore, we first compared its expression patterns in these two organs between young (4‐month‐old) and aged (22‐month‐old) mice. Western blot analysis revealed only a minimal change in hepatic ANGPTL8 expression with aging (Figure 6A, quantification shown in Figure S4A), whereas epididymal white adipose tissue exhibited a pronounced upregulation in aged mice (Figure 6B, quantification shown in Figure S4B), suggesting that adipose tissue is the major contributor to the age‐dependent rise in circulating ANGPTL8.

To further characterize the hepatic distribution of *Angptl8*, we performed spatial transcriptomics and single‐cell RNA‐sequencing analyzes of mouse liver. Spatial mapping revealed localized enrichment of *Angptl8* transcripts within lipid‐accumulated pericentral regions of aged livers (Figure 6C). However, single‐cell RNA‐seq analysis demonstrated that *Angptl8* expression in hepatocytes remained largely unchanged with age (Figure 6D,E), indicating that hepatic *Angptl8* upregulation is spatially restricted and unlikely to account for the systemic increase observed during aging.

In contrast, analysis of human single‐cell transcriptomic datasets from subcutaneous adipose tissue revealed a clear age‐dependent increase in both the proportion and expression intensity of *ANGPTL8*‐positive adipose lineage cells. To enable direct visualization and comparison of cellular composition between young and aged adipose tissue, cell‐type annotation was performed independently within each age group (Figure 6F,G). To further identify the cellular populations contributing to this increase, we examined adipose tissue single‐cell profiles at higher resolution. Consistent with previous studies, clustering analysis identified two mature adipocyte populations (Adip_1 and Adip_2) as well as a preadipocyte population (Pre_Ad) (Whytock et al. 2024). Notably, all three populations exhibited age‐associated enrichment of inflammatory pathways, suggesting that adipose aging is accompanied by a coordinated pro‐inflammatory transcriptional program spanning the adipocyte developmental continuum from progenitor cells to mature adipocytes. Within this framework, ANGPTL8 expression demonstrated an age‐dependent increase across Adip_1, Adip_2, and Pre_Ad populations (Figure 6H,I). The most pronounced increase was observed in Adip_1 cells, a population previously reported to be enriched for senescence‐associated and inflammatory gene signatures and to display preferential enrichment of the SenMayo gene set during aging (Whytock et al. 2024). Importantly, although the relative ANGPTL8 transcript level within Pre_Ad cells showed only modest changes, the proportion of ANGPTL8‐positive preadipocytes increased with age. Consistent with this observation, immunoblot analysis of primary preadipocytes isolated from 22‐month‐old mice demonstrated higher ANGPTL8 protein expression compared with cells isolated from young (4‐month‐old) mice (Figure S4C). Together, these results indicate that ANGPTL8 upregulation is not restricted to mature adipocytes but occurs throughout the adipocyte lineage during aging. Therefore, preadipocytes provide a relevant system to interrogate ANGPTL8‐driven adipose senescence at its cellular origin, enabling the investigation of how senescence programs are initiated and propagated along the adipocyte differentiation trajectory.

Given that visceral adipose tissue is particularly sensitive to aging and metabolic dysfunction (Muzumdar et al. 2008), and that our previous MRI‐based analyzes demonstrated that *Angptl8* deficiency attenuates age‐associated visceral fat accumulation, we next focused on epididymal white adipose tissue (eWAT) to assess whether ANGPTL8 directly contributes to adipose tissue senescence in vivo. Histological assessment of epididymal white adipose tissue (eWAT) revealed marked structural differences between genotypes in aged mice (Liu et al. 2025). Compared with age‐matched WT controls, adipocytes from *Angptl8* ^*−/−*^ mice were smaller in size and more densely organized (Figure 6J), suggesting attenuation of age‐related adipocyte hypertrophy and pathological adipose tissue remodeling. Consistent with these morphological changes, immunofluorescence analyzes demonstrated markedly reduced expression of the canonical senescence markers p16 and p21 in eWAT from aged *Angptl8* ^−/−^ mice compared with WT controls (Figure 6K). We next examined transcriptional senescence signatures in eWAT collected from Group 1 mice. As expected, mRNA levels of p16 and p21 increased with age in both WT and *Angptl8* ^*−/−*^ mice (Figure 6L). Notably, however, this age‐associated upregulation was significantly attenuated in *Angptl8* ^*−/−*^ mice, indicating delayed adipose tissue senescence upon loss of *Angptl8*. Consistent with the transcriptional findings, Western blot analyzes further confirmed reduced protein levels of p16 and p21 in eWAT from aged *Angptl8* ^*−/−*^ mice relative to age‐matched WT controls (Figure 6M, quantification shown in Figure S4D). To further validate adipose tissue senescence at the histological level, we performed senescence‐associated β‐galactosidase (SA‐β‐Gal) staining of adipose tissue sections. Aged WT mice exhibited a marked increase in SA‐β‐Gal‐positive cells compared with young controls, whereas this age‐associated accumulation was significantly attenuated in age‐matched *Angptl8* ^*−/−*^ mice (Figure S4E). Notably, crown‐like structures (CLS), a characteristic feature of adipose tissue inflammation and macrophage recruitment (Cinti et al. 2005), were also substantially reduced in aged *Angptl8* ^*−/−*^ mice. Consistent with these observations, immunofluorescence staining for the macrophage marker F4/80 demonstrated decreased macrophage infiltration in adipose tissue from aged *Angptl8* ^*−/−*^ mice relative to aged WT controls (Figure S4F), supporting a close association between ANGPTL8‐mediated adipose tissue senescence and inflammatory remodeling during aging.

Given the central role of senescent adipose tissue as a major contributor to chronic inflammation during aging, and the observed reductions in both adipose macrophage infiltration and CLS formation in *Angptl8*‐deficient mice, we next examined whether attenuation of adipose tissue senescence translated into reduced circulating SASP factors. Enzyme‐linked immunosorbent assay (ELISA) analysis revealed that multiple pro‐inflammatory SASP components were significantly elevated in the circulation of aged WT mice, whereas these age‐associated increases were markedly blunted in *Angptl8* ^*−/−*^ mice (Figure 6N). These findings indicate that loss of *Angptl8* not only alleviates adipose tissue senescence at the cellular and tissue levels but also limits the systemic accumulation of circulating SASP factors during aging. Collectively, our data support a model in which age‐associated upregulation of ANGPTL8 in adipose tissue promotes adipocyte senescence and SASP activation, thereby contributing to chronic low‐grade inflammation and systemic aging.

Collectively, these findings indicate that age‐associated upregulation of ANGPTL8 in adipose tissue promotes adipocyte senescence and SASP activation, thereby contributing to chronic low‐grade inflammation during aging. Importantly, the concordant reduction of canonical senescence markers in aged adipose tissue and in cultured preadipocytes derived from *Angptl8* ^*−/−*^ mice supports a direct, cell‐intrinsic role of ANGPTL8 in driving adipocyte senescence, rather than a secondary consequence of systemic aging. Importantly, building on our previous work demonstrating a direct intracellular interaction between ANGPTL8 and AKT2, we next sought to determine whether ANGPTL8 drives adipocyte senescence through activation of the AKT/mTOR signaling axis, a central pathway governing cellular growth, metabolism, and aging.

### 3.6 ANGPTL8 Activates the AKT/mTOR/S6K Axis to Drive Cellular Senescence

**FIGURE 7:**
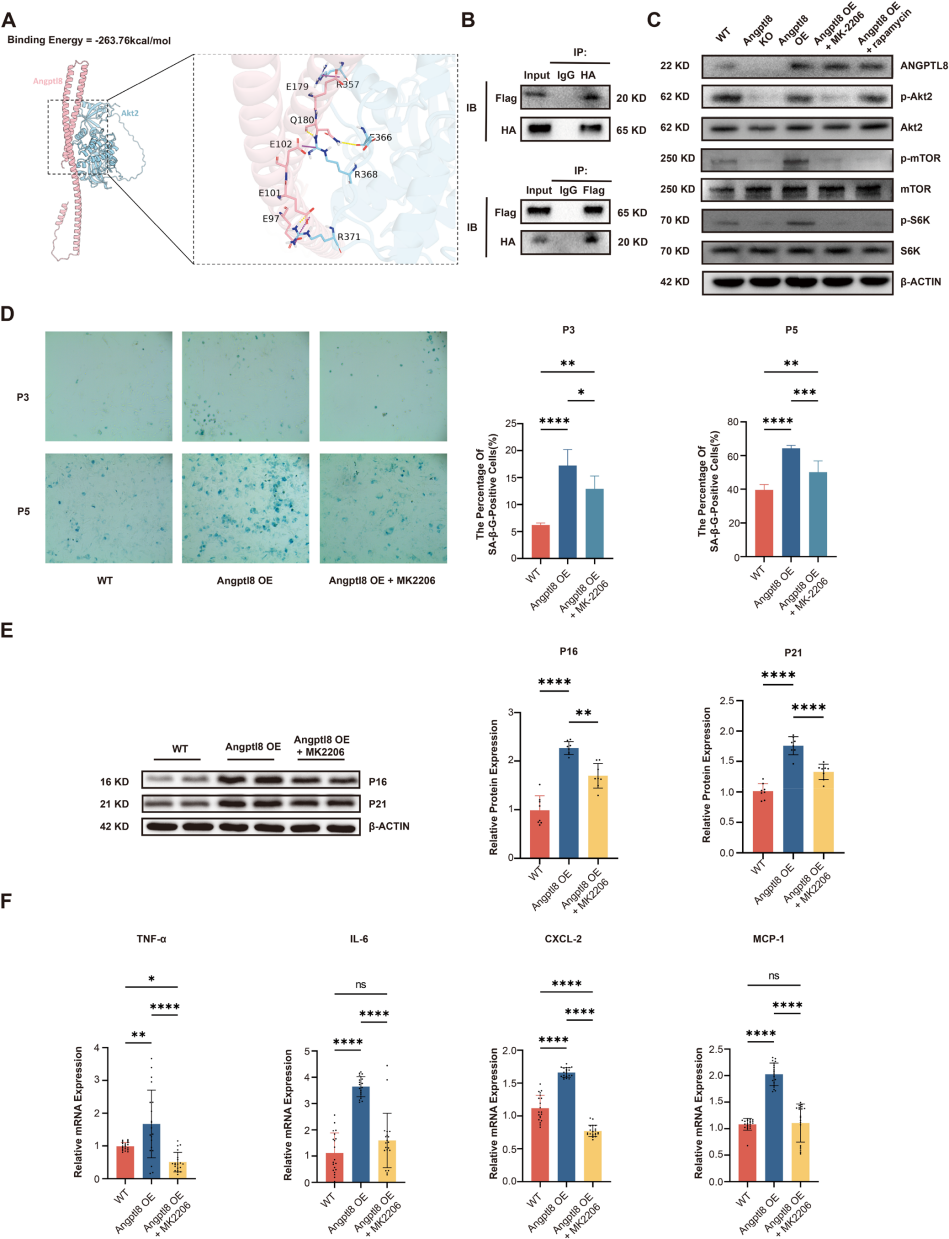
ANGPTL8 promotes adipocyte senescence through AKT2–mTOR–S6K signaling. (A) Predicted binding mode of the ANGPTL8–AKT2 complex. ANGPTL8 is shown in pink and AKT2 in blue. Yellow dashed lines indicate putative hydrogen bond interactions between the two proteins. Molecular docking predicted a potential interaction interface between ANGPTL8 and AKT2, with a docking score of approximately −263.76 kcal/mo. (B) Immunoprecipitation (IP) assays in undifferentiated 3 T3‐L1 preadipocytes co‐transfected with HA‐tagged ANGPTL8 and Flag‐tagged AKT2. Cell lysates were immunoprecipitated with anti‐HA or anti‐Flag antibodies, followed by immunoblotting with the indicated antibodies to assess protein–protein interactions. (C) Representative western blot images showing the expression and phosphorylation levels of AKT2, mTOR, and S6K in primary preadipocytes isolated from 4‐month‐old mice. Cells were assigned to five experimental groups: Wild‐type (WT), *Angptl8* ^*−/−*^ (*Angptl8* KO), *Angptl8* overexpression (*Angptl8* OE), *Angptl8* OE treated with the AKT inhibitor MK‐2206, and *Angptl8* OE treated with the mTOR inhibitor rapamycin. (D) Senescence‐associated β‐galactosidase (SA‐β‐gal) staining of epididymal white adipose tissue–derived primary cells from WT mice, *Angptl8*‐overexpressing (*Angptl8* OE) mice, and *Angptl8* OE primary cells treated with MK2206. SA‐β‐gal staining was performed at passage 3 (P3) and passage 5 (P5), where P3 and P5 denote the third and fifth passages of primary preadipocytes in culture, respectively. Data are presented as mean ± SEM. Normality was assessed using the Shapiro–Wilk test. Statistical significance was determined by one‐way ANOVA followed by Tukey's multiple‐comparisons test. ns, *p* > 0.05; *p < 0.05; **p < 0.01; ***p < 0.001; ****p < 0.0001. (E) Representative western blot images and quantitative analyzes of P16 and P21 protein expression in primary preadipocytes isolated from 4‐month‐old mice and cultured in vitro to passage 5 (P5). Cells were divided into three groups: WT, *Angptl8* OE, and *Angptl8* OE + MK‐2206. Quantitative data were obtained from eight biologically independent samples per group (*n* = 8). Data are presented as mean ± SEM. Normality was assessed using the Shapiro–Wilk test. Statistical significance was determined by one‐way ANOVA followed by Tukey's multiple‐comparisons test. ns, *p* > 0.05; *p < 0.05; **p < 0.01; ***p < 0.001; ****p < 0.0001. (F) Quantitative real‐time PCR analysis of senescence‐associated secretory phenotype (SASP) factors in primary preadipocytes from WT, *Angptl8* OE, and *Angptl8* OE + MK‐2206 groups. Quantitative analyzes were performed using ten biologically independent samples per group (*n* = 10). Data are presented as mean ± SEM. Normality was assessed using the Shapiro–Wilk test. Statistical significance was determined by one‐way ANOVA followed by Tukey's multiple‐comparisons test. ns, *p* > 0.05; *p < 0.05; **p < 0.01; ***p < 0.001; ****p < 0.0001.

Our previous work identified AKT2 as a direct binding partner of ANGPTL8 and demonstrated that this interaction mediates inflammatory and fibrotic responses in renal tubular cells (Pan et al. 2026). Building upon this finding, we hypothesized that the ANGPTL8–Akt2 interaction may not be restricted to inflammation, but instead represents a broader molecular module with context‐dependent functional consequences. Given the central role of AKT2 in coordinating signaling pathways governing cellular growth, metabolism, and survival, we further postulated that ANGPTL8 binding to Akt2 in adipocytes could influence aging‐related processes. To explore the potential structural basis underlying this interaction, we performed in silico molecular docking analyzes to predict the interaction interface between ANGPTL8 and AKT2. The docking analysis suggested a potential binding pose between ANGPTL8 and AKT2, with a predicted docking score of approximately −263.76 kcal/mol, providing computational structural support for the potential association between these proteins (Figure 7A).

Notably, the predicted binding interface localized to regulatory regions of AKT2 implicated in conformational control and kinase activation, providing a structural framework through which ANGPTL8 may modulate AKT2 signaling activity. Consistent with this notion, previous studies employing truncated AKT2 constructs have demonstrated that protein–protein interactions within these regulatory domains are sufficient to enhance AKT2 phosphorylation and downstream signaling activation. In this context, our docking results support a model in which intracellular binding of ANGPTL8 to AKT2 may facilitate AKT2 phosphorylation, thereby amplifying AKT2‐dependent signaling cascades.

To experimentally validate the predicted intracellular association between ANGPTL8 and AKT2, we performed co‐immunoprecipitation (co‐IP) assays in undifferentiated 3 T3‐L1 preadipocytes. As shown in Figure 7B, AKT2 was readily detected in ANGPTL8 immunoprecipitates, whereas no specific signal was observed in the corresponding IgG controls, indicating a specific interaction between ANGPTL8 and AKT2 within adipocytes. To further determine whether this interaction persists following adipocyte differentiation, we performed additional co‐IP assays in differentiated 3 T3‐L1 adipocytes and observed a similar association between ANGPTL8 and AKT2 (Figure S5A). These results provide biochemical evidence supporting the physical association of ANGPTL8 with AKT2 in a cellular context, thereby corroborating the in silico docking predictions. Importantly, the detection of this interaction in adipocytes suggests that ANGPTL8–AKT2 binding is not restricted to renal tubular cells, as previously reported, but may represent a more generalizable intracellular interaction with potential relevance to adipocyte biology and aging‐related signaling pathways.

To determine whether activation of the AKT/mTOR/S6K signaling pathway also occurs in vivo during adipose tissue aging, we first examined pathway activity in visceral adipose tissue homogenates obtained from young wild‐type (YG WT), old wild‐type (OD WT), and age‐matched *Angptl8* ^*−/−*^ mice. Western blot analysis revealed a marked age‐associated increase in the phosphorylation levels of AKT2, mTOR, and S6K in adipose tissue from old WT mice compared with young controls (Figure S5B). Importantly, genetic deletion of *Angptl8* significantly attenuated the activation of all three signaling components in aged adipose tissue, indicating that ANGPTL8 contributes to age‐related activation of the AKT/mTOR/S6K pathway in vivo.

To functionally determine whether ANGPTL8 promotes adipocyte senescence through the AKT/mTOR signaling axis, primary preadipocytes were isolated from 4‐month‐old mice and subjected to genetic and pharmacological manipulation. Cells from wild‐type (WT) mice served as controls, whereas preadipocytes isolated from *Angptl8* overexpression (*Angptl8* OE) mice were used to model enhanced ANGPTL8 activity. To further interrogate the signaling cascade, *Angptl8* OE preadipocytes were treated either with the AKT‐specific inhibitor MK2206 or with the mTOR inhibitor rapamycin. In parallel, preadipocytes isolated from *Angptl8* ^*−/−*^ mice were included to assess the effects of genetic loss of *Angptl8*. Consistent with our in vivo observations, *Angptl8* overexpression markedly increased AKT2 phosphorylation and was accompanied by enhanced activation of downstream mTOR and S6K signaling (Figure 7C, quantification shown in Figure S5E). In contrast, genetic deletion of *Angptl8* reduced the phosphorylation levels of AKT2, mTOR, and S6K. Pharmacological inhibition of AKT with MK2206 substantially attenuated AKT2 phosphorylation and downstream pathway activation, indicating that ANGPTL8‐mediated signaling is largely dependent on AKT activation. Consistent with these findings, treatment with rapamycin effectively suppressed the ANGPTL8‐induced increase in mTOR and S6K phosphorylation, further supporting a critical role for mTOR signaling downstream of ANGPTL8. Notably, total AKT2 and mTOR protein levels remained largely unchanged across experimental conditions, suggesting that ANGPTL8 primarily regulates pathway activity through phosphorylation‐dependent activation rather than alterations in protein abundance.

To assess the functional consequences of AKT activation on cellular senescence, primary preadipocytes were isolated from 22‐month‐old mice and serially passaged in vitro. Rather than using cells derived from young animals, we deliberately utilized aged donor mice to retain intrinsic aging‐associated features of adipose progenitor cells and thereby more closely recapitulate the physiological senescence state present in aged adipose tissue. Cells from each group were subsequently analyzed at passages 3 (P3) and 5 (P5) for senescence‐associated β‐galactosidase (SA‐β‐gal) activity. As shown in Figure 7D, *Angptl8* OE markedly increased the proportion of SA‐β‐gal–positive cells compared with control preadipocytes, indicating accelerated cellular senescence. Importantly, pharmacological inhibition of AKT signaling by MK2206 substantially attenuated the ANGPTL8‐induced senescent phenotype at both passages, demonstrating that AKT activity is required for ANGPTL8‐driven senescence in preadipocytes. To corroborate these phenotypic observations at the molecular level, protein extracts were prepared from *Angptl8* OE preadipocytes with or without MK2206 treatment and subjected to immunoblot analysis. Consistent with the SA‐β‐gal results, inhibition of AKT signaling by MK2206 markedly reduced the expression of both p16 and p21, indicating that suppression of AKT activity reverses ANGPTL8‐induced activation of senescence‐associated molecular programs. Given that cellular senescence is frequently accompanied by a pro‐inflammatory secretory program, we next examined the SASP at the transcriptional level. Using the same experimental design, total RNA was extracted from P5 preadipocytes, and representative SASP factors were quantified by quantitative PCR. As shown in Figure 7F, *Angptl8* OE induced a coordinated upregulation of multiple SASP‐related transcripts, indicating that ANGPTL8‐driven senescence is associated with enhanced inflammatory gene expression. Notably, AKT inhibition by MK2206 markedly suppressed the ANGPTL8‐driven transcriptional activation of these SASP factors, further supporting an AKT‐dependent mechanism.

To further address whether the pro‐senescent effects of ANGPTL8 extend beyond preadipocytes and persist following adipocyte differentiation, primary preadipocytes isolated from 4‐month‐old WT and *Angptl8* ^*−/−*^ mice were induced to differentiate into mature adipocytes in vitro. Consistent with our observations in aged adipose tissue, ANGPTL8 deficiency significantly reduced the secretion of SASP factors in differentiated adipocytes (Figure S5C). Moreover, quantitative PCR analysis demonstrated significantly lower expression of the senescence markers p16 and p21 in mature adipocytes derived from *Angptl8* ^*−/−*^ mice compared with WT controls (Figure S5D). These findings indicate that the pro‐senescent effects of ANGPTL8 are maintained after adipocyte differentiation and support a role for ANGPTL8 in promoting senescence throughout adipocyte maturation rather than acting exclusively at the preadipocyte stage.

Collectively, these molecular, cellular, and functional analyzes delineate a coherent mechanistic framework in which intracellular ANGPTL8 interacts with AKT2, enhances AKT/mTOR/S6K signaling, and thereby promotes adipocyte senescence and the acquisition of a pro‐inflammatory SASP phenotype. The consistent attenuation of senescence markers, SASP factor production, and pathway activation following genetic ablation of *Angptl8* or pharmacological inhibition of AKT signaling supports a causal role for ANGPTL8–AKT2 signaling in driving adipose tissue aging. Taken together with the age‐associated enrichment of ANGPTL8 in adipose tissue and its contribution to circulating SASP factors, these findings position ANGPTL8 as a previously unrecognized intracellular regulator linking adipocyte‐intrinsic senescence programs to systemic inflammaging during aging.

## 4 Discussion

Aging is increasingly recognized as a chronic, low‐grade inflammatory state driven by sustained cytokine production and persistent immune activation, a phenomenon commonly referred to as inflammaging (Franceschi et al. 2000; Franceschi et al. 2018). Accumulating evidence indicates that this inflammatory milieu is largely fueled by the progressive accumulation of senescent cells, which secrete a complex array of pro‐inflammatory cytokines, chemokines, growth factors, and proteases collectively termed the senescence‐associated secretory phenotype (SASP) (Campisi and d'Adda di Fagagna 2007; Coppé et al. 2010). Among peripheral tissues, adipose tissue has emerged as a dominant contributor to age‐associated systemic inflammation. Owing to its substantial mass, early susceptibility to cellular senescence, and robust secretory capacity, aging adipose tissue acts as a major source of circulating SASP factors that propagate inflammatory signals and exacerbate organismal aging (Islam et al. 2023; Xu et al. 2015). These observations support the concept that adipose tissue is not merely a passive energy reservoir, but rather a central endocrine and immunometabolic organ that actively shapes the trajectory of aging.

Through an integrated framework combining population‐scale analyzes, genetic mouse models, and mechanistic cellular investigations, we identify ANGPTL8 as an adipose‐derived regulator that couples age‐associated adipose tissue dysfunction to systemic aging phenotypes. Circulating ANGPTL8 levels increased progressively with chronological aging in both humans and mice, and population‐based machine learning analyzes revealed a robust association between ANGPTL8 abundance, biological aging trajectories, and mortality risk. While these human data establish ANGPTL8 as a clinically relevant aging‐associated biomarker, they do not independently define a causal relationship. Complementing these observations, genetic ablation of *Angptl8* in mice provided functional evidence that ANGPTL8 actively contributes to the aging process, as its deficiency extended lifespan, attenuated multi‐organ aging phenotypes, preserved skeletal muscle integrity, and improved physical performance in aged animals. Collectively, our findings position ANGPTL8 as a previously underappreciated molecular link within the adipose–systemic aging axis, transforming the view of ANGPTL8 from a metabolic biomarker associated with aging to a functional mediator capable of modulating organismal aging trajectories.

A central advance of this study lies in defining the tissue origin of age‐associated ANGPTL8 elevation. Although ANGPTL8 is classically considered a hepatokine, re‐analysis of published spatial transcriptomic and single‐cell RNA sequencing datasets revealed that hepatic *Angptl8* expression exhibits only modest and spatially restricted changes with aging (Nikopoulou et al. 2023). In contrast, both murine and human adipose tissues displayed a pronounced age‐dependent upregulation of *ANGPTL8*, with increased expression particularly evident within adipocyte subpopulations previously annotated as inflammation‐ and senescence‐associated clusters (Whytock et al. 2024). This cross‐species concordance strongly supports adipose tissue as the predominant source of elevated circulating ANGPTL8 during aging and reinforces the concept that adipose tissue functions as an active endocrine driver, rather than a passive bystander, in the aging process.

Beyond its systemic endocrine role, our in vivo and in vitro experiments demonstrate that ANGPTL8 exerts a direct, cell‐autonomous pro‐senescent effect within adipocytes. Angptl8 deficiency markedly attenuated the age‐associated induction of canonical senescence markers p16 and p21, limited adipocyte hypertrophy, and preserved adipose tissue architecture. Consistently, primary preadipocytes derived from *Angptl8* ^*−/−*^ mice exhibited delayed replicative senescence and reduced SA‐β‐Gal positivity. These findings establish ANGPTL8 as an intrinsic regulator of adipocyte aging, rather than a secondary consequence of systemic metabolic alterations. Given the endocrine nature of adipose tissue, such cell‐autonomous senescence is well positioned to amplify organism‐wide aging signals through SASP‐mediated mechanisms.

Mechanistically, we identify AKT2 as a direct intracellular binding partner of ANGPTL8, providing a molecular basis for its pro‐senescent activity. The AKT–mTOR–S6K axis is a central nutrient‐ and growth factor–sensing pathway, whose chronic activation has been causally linked to cellular senescence, tissue degeneration, and organismal aging (Harrison et al. 2009; Laberge et al. 2015; Panwar et al. 2023; Zoncu et al. 2011). We show that ANGPTL8 binding enhances AKT2 phosphorylation and promotes downstream activation of mTOR and S6K signaling. Importantly, pharmacological blockade of AKT with MK2206, direct inhibition of mTOR with rapamycin, or genetic disruption of *Angptl8* effectively suppressed mTOR/S6K activation and attenuated ANGPTL8‐induced senescence phenotypes, establishing the AKT–mTOR signaling cascade as a functionally required mediator of ANGPTL8‐driven adipocyte senescence. Collectively, these data expand the biological repertoire of ANGPTL8 beyond lipid metabolism and place it within a conserved aging‐associated signaling pathway that couples intracellular metabolic sensing to adipocyte senescence and inflammatory output.

Importantly, the systemic consequences of ANGPTL8‐driven adipose aging are most parsimoniously explained by the amplification of senescence‐associated secretory phenotype signaling. Aging adipose tissue represents a major source of chronic, low‐grade inflammatory mediators, and ANGPTL8‐driven adipocyte senescence markedly enhances this proinflammatory output. Such sustained SASP signaling provides a plausible mechanistic link between adipose tissue dysfunction and the progressive decline of organismal physiological functions observed during aging. In this framework, the preservation of physical performance and tissue integrity in *Angptl8*‐deficient mice is consistent with attenuation of adipose‐derived inflammatory stress, rather than tissue‐specific effects in distal organs.

Given that adipose tissue is among the earliest metabolic organs to undergo age‐related decline, its dysfunction may serve as a critical upstream driver of systemic inflammaging. Through impaired lipid handling, increased SASP production, and chronic inflammatory activation, senescent adipose tissue can disrupt whole‐body homeostasis and promote frailty. By focusing on adipocyte‐intrinsic ANGPTL8, our study shifts attention away from a liver‐centric view of ANGPTL8 biology and highlights adipose tissue aging as a central pathogenic node. This perspective not only reconciles circulating biomarker data with tissue‐level mechanisms but also underscores the importance of targeting adipose‐derived signals in aging interventions.

From a translational standpoint, the dual identity of ANGPTL8 as both a circulating biomarker and a functional effector of aging is particularly compelling. Our MBA8‐Clock and AIMR‐Model analyzes demonstrate that ANGPTL8 robustly predicts biological age and mortality risk, outperforming many conventional clinical parameters. Beyond its association with biological age and mortality risk, we further observed that higher circulating ANGPTL8 levels were associated with increased frailty and adverse visceral metabolic phenotypes in older adults, extending the clinical relevance of ANGPTL8 to functional and metabolic manifestations of aging. Notably, our animal studies also suggested that *Angptl8* deficiency attenuated age‐related muscle loss, raising the possibility that ANGPTL8 may contribute to musculoskeletal decline indirectly through adipose tissue senescence and chronic inflammatory remodeling. However, our current human cohort did not include muscle‐related measurements, which precluded further evaluation of whether the association between ANGPTL8 and age‐related muscle loss observed in our animal studies could also be detected at the population level. Future studies incorporating comprehensive assessments of body composition, muscle function, and frailty will be important to determine whether the effects of ANGPTL8 on aging extend beyond adipose tissue to the musculoskeletal system. Moreover, therapeutic strategies aimed at neutralizing ANGPTL8 or disrupting its intracellular interaction with AKT2 may represent viable approaches to delay adipose senescence, dampen systemic inflammation, and preserve physical function during aging.

Several limitations merit consideration. Although the MBA8‐Clock demonstrated robust predictive performance, the contribution of certain lifestyle‐related variables should be interpreted with caution. For example, smoking status was not consistently associated with accelerated biological aging in the expected direction within our cohort. This observation may reflect survivor bias inherent to older community‐based populations, limitations in the granularity of smoking exposure data, or interactions with other metabolic and demographic variables incorporated into the machine‐learning framework. While our data strongly implicate adipose tissue as the dominant source and target of ANGPTL8 during aging, contributions from other metabolic organs cannot be fully excluded. In addition, although we establish a mechanistic requirement for AKT2–mTOR signaling in ANGPTL8‐mediated senescence, future studies employing adipocyte‐specific genetic models or neutralizing antibodies will be necessary to determine the organismal efficacy of targeting this pathway. Finally, given the sensitivity of ANGPTL8 to nutritional and metabolic cues, it will be important to explore how dietary interventions intersect with ANGPTL8‐driven aging processes.

In conclusion, this study identifies ANGPTL8 as a pro‐senescent adipokine that links adipose tissue dysfunction to systemic aging through an AKT2–mTOR–SASP axis. By integrating human predictive modeling with in vivo validation and mechanistic interrogation, our work provides a conceptual framework connecting metabolic regulation, adipocyte senescence, inflammaging, and functional decline. These findings not only advance our understanding of aging biology but also position ANGPTL8 as a promising biomarker and therapeutic target for age‐associated disorders.

## Author Contributions

**Yi He:** conceptualization, methodology, visualization, writing – review and editing, writing – original draft. **Limeng Pan:** methodology, resources, writing – original draft. **Wenjun Ping:** methodology. **Chen Meng:** methodology. **Xiaoyu Meng:** software. **Yuxi Xiang:** methodology, validation. **Beibei Mao:** methodology, validation. **Xiaoyu Meng:** formal analysis, investigation, methodology. **Yaming Guo:** methodology, resources. **Ranran Kan:** methodology, validation. **Siyi Wang:** methodology, software. **Danpei Li** and **Xuefeng Yu:** supervised the study and contributed to the writing, review, and editing of the manuscript.

## Funding

This work was supported by National Natural Science Foundation of China (Grants 82270880, 82470907 and 82400944).

## Ethics Statement

Human studies were approved by the Ethics Committees of Ruijin Hospital, Shanghai Jiao Tong University School of Medicine (No. 14/2004) and Tongji Hospital, Huazhong University of Science and Technology (No. TJ‐IRB20231125), and written informed consent was obtained from all participants. Animal experiments were approved by the Institutional Animal Care and Use Committee of Tongji Hospital, Huazhong University of Science and Technology (No. TJH‐202303011) and conducted in accordance with relevant guidelines and regulations.

## Conflicts of Interest

The authors declare no conflicts of interest.

## Supporting information


**Figure S1:** Screening of key predictive features for model construction via multicollinearity analysis and Boruta‐LASSO algorithm. (A) No pairwise variable correlations exceeded the preset multicollinearity threshold, indicating no severe multicollinearity among the candidate features. All variables were retained for subsequent feature screening to avoid the exclusion of potential predictive factors. (B–D) The hybrid Boruta–LASSO screening procedure was applied to filter critical predictive fea‐tures for the construction of the MBA8‐Clock and AIMR model. A total of 22 independent key predictors were finally identified, covering biochemical indicators (ANGPTL8, HbA1c, Insulin, FBG, PBG, Creatinine, HDL‐C, LDL‐C, TC, TG, ALT, AST, GGT), physiological indicators (SBP, DBP, Pulse, Weight, Waist circumference, Hip circumference), and lifestyle factors (Snore, Smoking, Exercise). These screened key features were subsequently used to develop the MBA8‐Clock and AIMR model based on five machine learning classifiers, including Random Forest, Neural Network, LightGBM, Multilayer Perceptron, and XGBoost.

**Figure S2:** Association of circulating ANGPTL8 levels with aging‐related clinical phenotypes in the 4C cohort. (A) Frailty index across quartiles of circulating ANGPTL8 concentrations. Participants were stratified into quartiles according to serum ANGPTL8 levels (Q1–Q4). (B) Metabolic Score for Visceral Fat (METS‐VF) across ANGPTL8 quartiles. Data are presented as mean ± SEM. Differences among groups were analyzed by one‐way ANOVA, and trend analyzes were performed across ANGPTL8 quartiles.

**Figure S3:** acel70671‐sup‐0003‐FigureS3.pdf. *Angptl8* deficiency regulates adipose tissue distribution and mass in aged mice. (A) Bar graphs showing adipose tissue mass as a percentage of total body weight in 22‐month‐old wild‐type (WT) mice (*n* = 5) and age‐matched *Angptl8* ^*−/−*^ mice (*n* = 5). Adipose tissue mass was calculated as the combined weight of epididymal and inguinal fat depots. Statistical comparisons between groups were performed using an unpaired two‐tailed Student's *t*‐test. Data are presented as mean ± SEM. ns, *p* > 0.05. (B) Bar graphs showing weights of subcutaneous (inguinal) and visceral (epididymal) fat pads in 22‐month‐old wild‐type (WT) mice (*n* = 5) and age‐matched *Angptl8* ^*−/−*^ mice (*n* = 5). Statistical comparisons between groups were performed using unpaired two‐tailed Student's *t*‐tests. Data are presented as mean ± SEM. ns, *p* > 0.05; *p < 0.05.

**Figure S4:** acel70671‐sup‐0004‐FigureS4.pdf. *Angptl8* deficiency attenuates adipose tissue senescence and inflammatory remodeling during aging. (A) ANGPTL8 protein expression in liver tissues collected from young wild‐type (YG WT, 4‐month‐old) and old wild‐type (OD WT, 22‐month‐old) mice (*n* = 6 per group). Data are presented as mean ± SEM. Statistical significance was assessed using an unpaired two‐tailed Student's *t*‐test. ns, *p* > 0.05. (B) ANGPTL8 protein expression in epididymal white adipose tissue (eWAT) collected from young wild‐type (YG WT, 4‐month‐old) and old wild‐type (OD WT, 22‐month‐old) mice (*n* = 6 per group). Data are presented as mean ± SEM. Statistical significance was assessed using an unpaired two‐tailed Student's *t*‐test. ns, *p* > 0.05; *p < 0.05; **p < 0.01; ***p < 0.001; ****p < 0.0001. (C) Representative western blot images and quantitative analysis of ANGPTL8 protein expression in primary preadipocytes isolated from inguinal white adipose tissue (iWAT) and epididymal white adipose tissue (eWAT) of young wild‐type (YG, 4‐month‐old) and old wild‐type (OD, 22‐month‐old) mice. The four groups analyzed were YG iWAT, YG eWAT, OD iWAT, and OD eWAT. ANGPTL8 protein abundance was increased in preadipocytes derived from aged adipose tissue compared with young controls. Data are presented as mean ± SEM from three biologically independent mice per group (*n* = 3). Due to the limited sample size, statistical significance was assessed using the Kruskal–Wallis test followed by Dunn's multiple‐comparisons test. ns, *p* > 0.05; *p < 0.05; **p < 0.01; ***p < 0.001. (D) Densitometric quantification of p16 and p21 protein expression in epididymal white adipose tissue (eWAT) collected from old wild‐type (OD WT, 22‐month‐old) and old *Angptl8* ^*−/−*^ (OD KO, 22‐month‐old) mice. Data are presented as mean ± SEM from six biologically independent mice per group (*n* = 6). Normality was assessed using the Shapiro–Wilk test. Statistical significance was determined using an unpaired two‐tailed Student's *t*‐test. ns, *p* > 0.05; *p < 0.05; **p < 0.01; ***p < 0.001; **p < 0.0001. (E) Representative senescence‐associated β‐galactosidase (SA‐β‐Gal) staining of epididymal adipose tissue sections from young wild‐type (YG WT), old wild‐type (OD WT), and old *Angptl8* ^*−/−*^ (OD KO) mice. Blue arrows indicate SA‐β‐Gal‐positive cells, and red arrows indicate crown‐like structures (CLS). Scale bar = 20 μm. Quantification of SA‐β‐Gal‐positive cells and CLS numbers is shown below. ns, *p* > 0.05; *p < 0.05; **p < 0.01; ***p < 0.001, ****p < 0.0001. (F) Representative immunofluorescence staining of the macrophage marker F4/80 in epididymal adipose tissue sections from YG WT, OD WT, and OD *Angptl8* ^*−/−*^ (OD KO) mice. F4/80 is shown in red and nuclei were counterstained with DAPI (blue). Scale bar = 50 μm. Quantification of relative F4/80 fluorescence intensity is shown below. ns, *p* > 0.05; *p < 0.05; **p < 0.01; ***p < 0.001, ****p < 0.0001.

**Figure S5:** Angptl8 interacts with AKT2 and mediates age‐dependent AKT/mTOR/S6K signaling activation in adipose tissue. (A) Validation of the interaction between ANGPTL8 and AKT2 in adipocytes. Immunoprecipitation (IP) analysis was performed in differentiated mature 3T3‐L1 adipocytes co‐transfected with HA‐tagged ANGPTL8 and Flag‐tagged AKT2. Cell lysates were immunoprecipitated using anti‐HA or anti‐Flag antibodies, followed by immunoblotting with specific antibodies to verify the protein–protein interaction between ANGPTL8 and AKT2. (B) *Angptl8* deficiency suppresses age‐induced activation of the AKT/mTOR/S6K signaling pathway in epididymal white adipose tissue. Representative western blot images displaying the total protein expression and phosphorylation levels of AKT2, mTOR, and S6K in visceral adipose tissue derived from young wild‐type (YG WT), aged wild‐type (OD WT), and aged *Angptl8* ^*−/−*^ (OD KO) mice. Data are presented as mean ± SEM (*n* = 3 biologically independent mice per group). Statistical significance was determined by Kruskal–Wallis test followed by Dunn's multiple‐comparisons test. *p* < 0.05 was considered statistically significant. **p < 0.01; ***p < 0.001. (C) Quantification of senescence‐associated secretory phenotype (SASP) factors in conditioned media collected from fully differentiated mature adipocytes derived from primary preadipocytes isolated from 4‐month‐old wild‐type (WT) and *Angptl8* ^*−/−*^ mice. Data are presented as mean ± SEM from eight biologically independent samples per group (*n* = 8). Normality was assessed using the Shapiro–Wilk test. Statistical significance was determined using an unpaired two‐tailed Student's *t*‐test. ns, *p* > 0.05; *p < 0.05; **p < 0.01; ***p < 0.001, ****p < 0.0001. (D) Quantitative real‐time PCR analysis of the senescence markers p16 and p21 in fully differentiated mature adipocytes derived from WT and *Angptl8* ^*−/−*^ primary preadipocytes. Data are presented as mean ± SEM from six biologically independent samples per group (*n* = 6). Normality was assessed using the Shapiro–Wilk test. Statistical significance was determined using an unpaired two‐tailed Student's *t*‐test. ns, *p* > 0.05; *p < 0.05; **p < 0.01; ***p < 0.001, ****p < 0.0001. (E) The expression and phosphorylation levels of AKT2, mTOR, and S6K in primary preadipocytes isolated from 4‐month‐old mice. Cells were assigned to five experimental groups: WT, *Angptl8* ^*−/−*^ (*Angptl8* KO), *Angptl8* overexpression (*Angptl8* OE), *Angptl8* OE treated with the AKT inhibitor MK‐2206, and *Angptl8* OE treated with the mTOR inhibitor rapamycin. Data are presented as mean ± SEM from eight biologically independent samples per group (*n* = 8). Normality was assessed using the Shapiro–Wilk test. Statistical significance was determined by one‐way ANOVA followed by Tukey's multiple‐comparisons test for multi‐ple‐group comparisons. ns, *p* > 0.05; *p < 0.05, **p < 0.01; ***p < 0.001, ****p < 0.0001.

**Table S1:** acel70671‐sup‐0006‐TableS1.xlsx.

**Table S2:** Primer sequences used for quantitative real‐time PCR analysis.

**Table S3:** Clinical characteristics of representative participants selected for MBA8‐clock interpretation.

**Table S4:** Clinical characteristics of representative participants selected for AIMR‐model interpretation.

**Table S5:** Final selected variables, model coefficients, and intercepts of the MBA8‐clock and AIMR‐model.

**Table S6:** Health deficits included in the frailty index (FI) calculation.

## Data Availability

The data that support the findings of this study are available on request from the corresponding author. The data are not publicly available due to privacy or ethical restrictions.
