# Supplementary material for: An ANGPTL8‐AKT2‐mTOR Axis Drives Adipose Senescence and Aging‐Related Functional Decline
Source: Aging Cell. 2026 Aug 16;25(8):e70671. doi: 10.1111/acel.70671 (PMC13478719; doi:10.1111/acel.70671)
Supplement: Supplementary file 1 [file ACEL-25-e70671-s001.pdf]

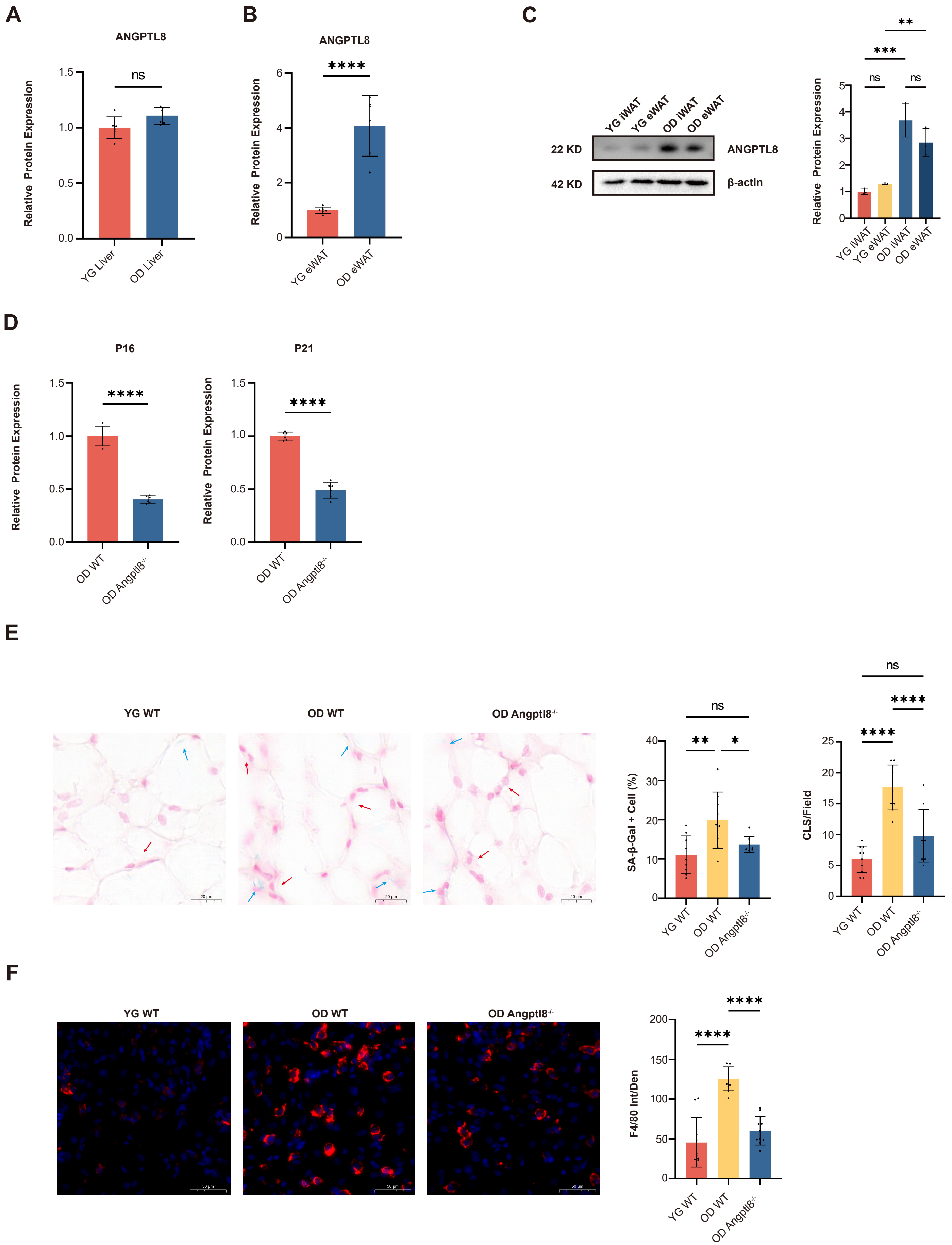

A
ANGPTL8
Relative Protein Expression
YG Liver
OD Liver
ns
B
ANGPTL8
Relative Protein Expression
YG eWAT
OD eWAT
****
C
YG iWAT
YG eWAT
OD iWAT
OD eWAT
22 KD
ANGPTL8
42 KD
β-actin
Relative Protein Expression
D
P16
P21
Relative Protein Expression
OD WT
OD Angptl8-/-
E
YG WT
OD WT
OD Angptl8-/-
SA-β-Gal + Cell (%)
CLS/Field
F
YG WT
OD WT
OD Angptl8-/-
F4/80 Int/Den
