# Supplementary material for: An ANGPTL8‐AKT2‐mTOR Axis Drives Adipose Senescence and Aging‐Related Functional Decline
Source: Aging Cell. 2026 Aug 16;25(8):e70671. doi: 10.1111/acel.70671 (PMC13478719; doi:10.1111/acel.70671)
Supplement: Supplementary file 2 [file ACEL-25-e70671-s003.pdf]

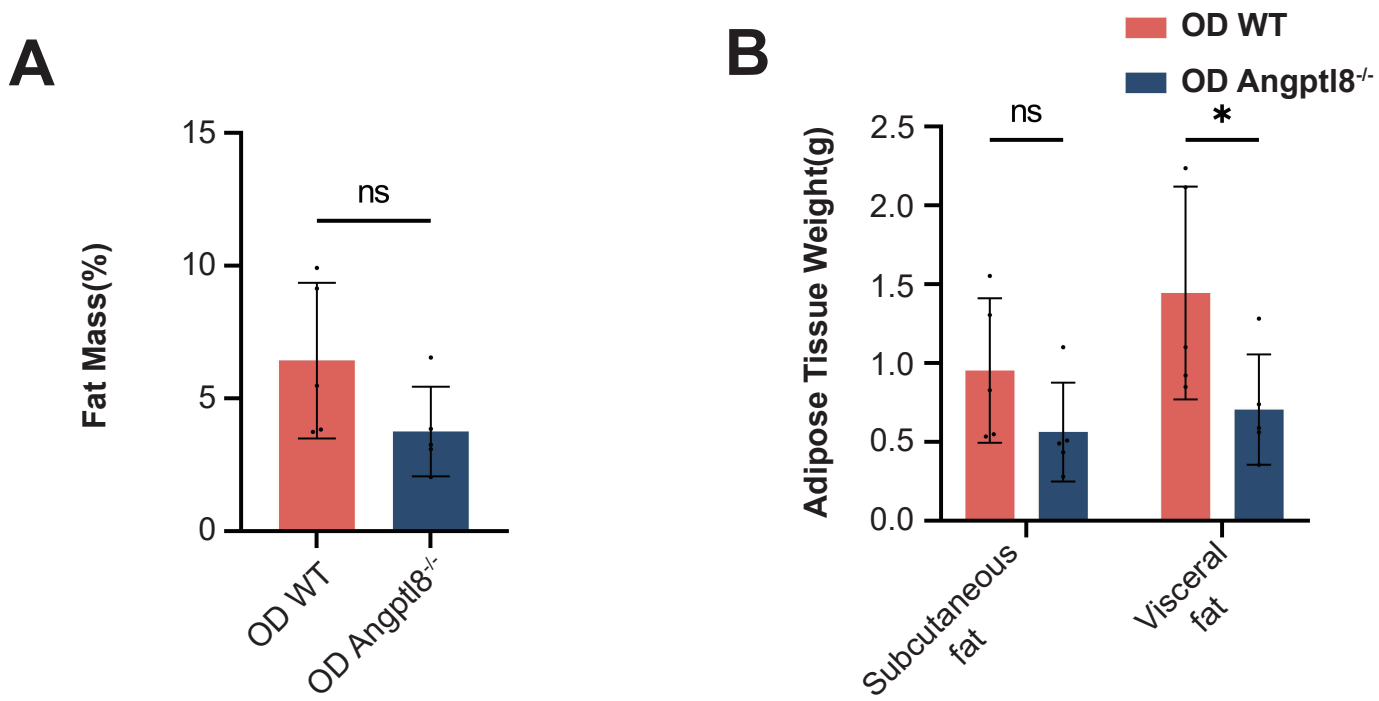
A
Fat Mass(%)
15
10
5
0
ns
OD WT
OD Angptl8-/-
B
OD WT
OD Angptl8-/-
Adipose Tissue Weight(g)
2.5
2.0
1.5
1.0
0.5
0.0
ns
*
Subcutaneous fat
Visceral fat
