# Supplementary material for: An ANGPTL8‐AKT2‐mTOR Axis Drives Adipose Senescence and Aging‐Related Functional Decline
Source: Aging Cell. 2026 Aug 16;25(8):e70671. doi: 10.1111/acel.70671 (PMC13478719; doi:10.1111/acel.70671)
Supplement: Supplementary file 3 [file ACEL-25-e70671-s008.pdf]

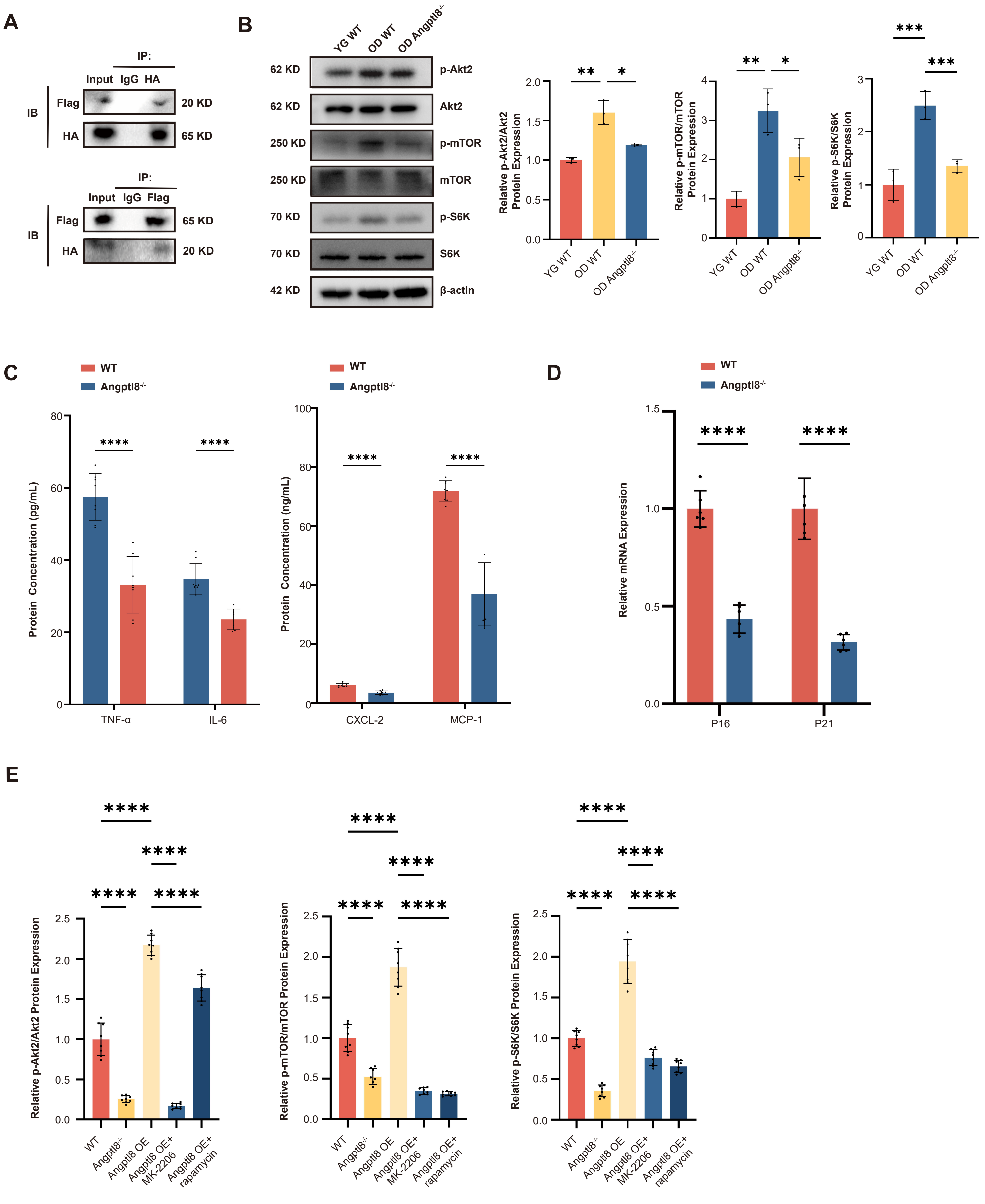

A
IP:
Input IgG HA
IB
Flag
HA
20 KD
65 KD
IP:
Input IgG Flag
IB
Flag
HA
65 KD
20 KD
B
YG WT
OD WT
OD Angptl8-/-
62 KD
p-Akt2
62 KD
Akt2
250 KD
p-mTOR
250 KD
mTOR
70 KD
p-S6K
70 KD
S6K
42 KD
β-actin
Relative p-Akt2/Akt2 Protein Expression
Relative p-mTOR/mTOR Protein Expression
Relative p-S6K/S6K Protein Expression
**
*
***
C
WT
Angptl8-/-
Protein Concentration (pg/mL)
TNF-α
IL-6
Protein Concentration (ng/mL)
CXCL-2
MCP-1
****
D
WT
Angptl8-/-
Relative mRNA Expression
P16
P21
****
E
Relative p-Akt2/Akt2 Protein Expression
Relative p-mTOR/mTOR Protein Expression
Relative p-S6K/S6K Protein Expression
WT
Angptl8-/-
Angptl8 OE
Angptl8 OE+ MK-2206
Angptl8 OE+ rapamycin
****
