# Supplementary material for: An ANGPTL8‐AKT2‐mTOR Axis Drives Adipose Senescence and Aging‐Related Functional Decline
Source: Aging Cell. 2026 Aug 16;25(8):e70671. doi: 10.1111/acel.70671 (PMC13478719; doi:10.1111/acel.70671)
Supplement: Supplementary file 4 [file ACEL-25-e70671-s010.pdf]

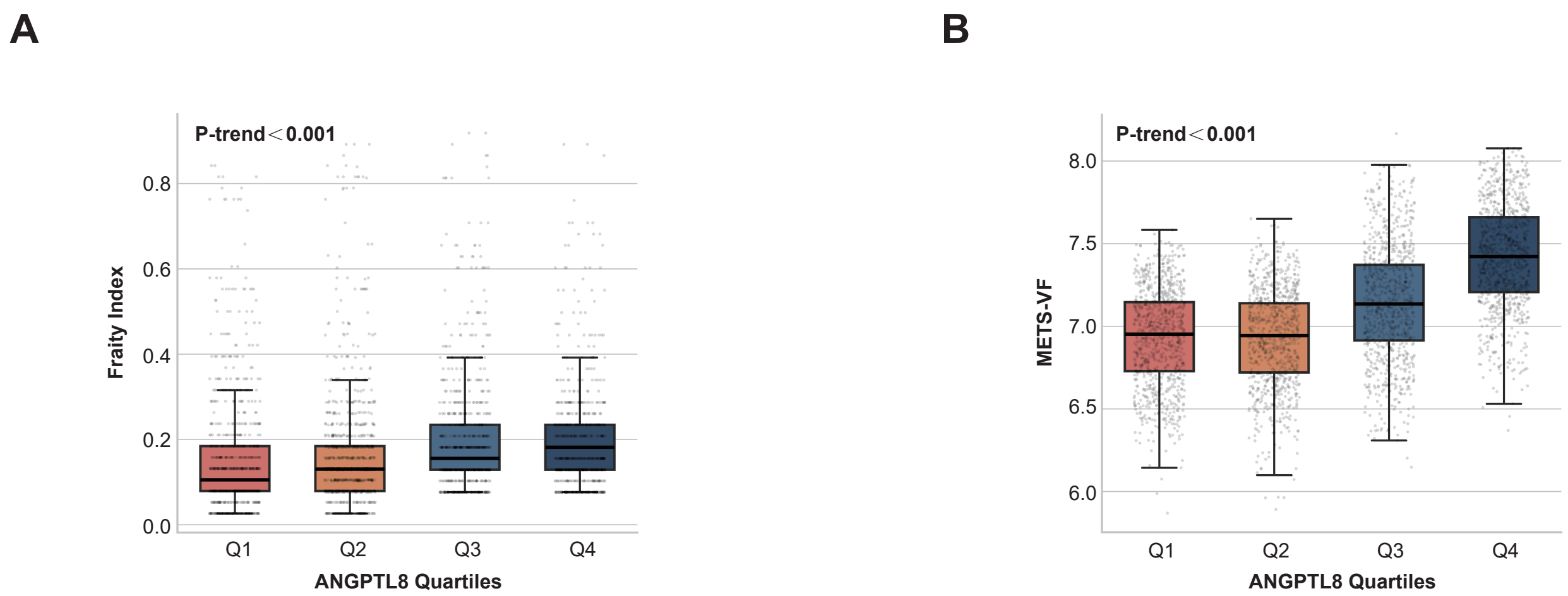

A
P-trend < 0.001
Fraity Index
0.0
0.2
0.4
0.6
0.8
Q1
Q2
Q3
Q4
ANGPTL8 Quartiles
B
P-trend < 0.001
METS-VF
6.0
6.5
7.0
7.5
8.0
Q1
Q2
Q3
Q4
ANGPTL8 Quartiles
