# Supplementary material for: An ANGPTL8‐AKT2‐mTOR Axis Drives Adipose Senescence and Aging‐Related Functional Decline
Source: Aging Cell. 2026 Aug 16;25(8):e70671. doi: 10.1111/acel.70671 (PMC13478719; doi:10.1111/acel.70671)
Supplement: Supplementary file 5 [file ACEL-25-e70671-s004.docx]

| Gene | Organisms | Primer Sequence 5’ to 3’ | |
| --- | --- | --- | --- |
|  |  | Forward | Reverse |
| *β-actin* | *Homo sapiens* | TCCCTGGAGAAGAGCTACGA | TGAAGGTAGTTTCGTGGATGC |
| *Angptl8* | *Homo sapiens* | GCCGCACAATAGAACTCCTG | CAAATTCTCGGTAGGCAGGG |
| *β-actin* | *Mus musculus* | GGCTGTATTCCCCTCCATCG | CCAGTTGGTAACAATGCCATGT |
| *Angptl8* | *Mus musculus* | ACCAGCCTGTCGGAGATTCA | TGGCCAGTGAGAGCCCATAA |
| *p16^Ink4a* | *Mus musculus* | CGCAGGTTCTTGGTCACTGT | TGTTCACGAAAGCCAGAGCG |
| *p21^Cip1* | *Mus musculus* | CCTGGTGATGTCCGACCTG | CCATGAGCGCATCGCAATC |
|  |  |  |  |
