# Supplementary material for: An ANGPTL8‐AKT2‐mTOR Axis Drives Adipose Senescence and Aging‐Related Functional Decline
Source: Aging Cell. 2026 Aug 16;25(8):e70671. doi: 10.1111/acel.70671 (PMC13478719; doi:10.1111/acel.70671)
Supplement: Supplementary file 6 [file ACEL-25-e70671-s005.docx]

|  | **Definition according to baseline self-report, physical measurements, or both** |  |
| --- | --- | --- |
| 1 | Self-reported diagnosis of hypertension by a doctor, self-reported use of antihypertension drugs, systolic blood pressure measured to be ≥140 mm Hg, or diastolic blood pressure measured to be ≥90 mm Hg | Yes=1·00; no=0·00 |
| 2 | Self-reported diagnosis of heart disease by a doctor | Yes=1·00; no=0·00 |
| 3 | Self-reported diagnosis of stroke or transient ischaemic attack by a doctor | Yes=1·00; no=0·00 |
| 4 | Self-reported diagnosis of emphysema or chronic bronchitis by a doctor | Yes=1·00; no=0·00 |
| 5 | Self-reported diagnosis of tuberculosis by a doctor | Yes=1·00; no=0·00 |
| 6 | Self-reported diagnosis of asthma by a doctor | Yes=1·00; no=0·00 |
| 7 | Self-reported diagnosis of peptic ulcer by a doctor | Yes=1·00; no=0·00 |
| 8 | Self-reported diagnosis of gallstone disease, with or without cholecystitis, by a doctor | Yes=1·00; no=0·00 |
| 9 | Self-reported diagnosis of rheumatoid arthritis by a doctor | Yes=1·00; no=0·00 |
| 10 | Self-reported diagnosis of fracture by a doctor | Yes=1·00; no=0·00 |
| 11 | Self-reported diagnosis of neurasthenia by a doctor | Yes=1·00; no=0·00 |
| 12 | Self-reported diagnosis of diabetes, fasting blood glucose measured to be ≥7·0 mmol/L, or random blood glucose measured to be ≥11·1 mmol/L | Yes=1·00; no=0·00 |
| 13 | Self-reported diagnosis of cancer by a doctor | Yes=1·00; no=0·00 |
| 14 | Self-reported diagnosis of chronic kidney disease by a doctor | Yes=1·00; no=0·00 |
| 15 | If you were walking on level ground with other healthy people of the same age, would you usually become short of breath or slow down because of chest discomfort? | Yes=1·00; no=0·00 |
| 16 | During the past month, did you have any of the following for ≥3 days per week: (1) taking >30 min to fall asleep after going to bed or waking up in the middle of the night; (2) waking up early and not being able to go back to sleep; or (3) having difficulty staying alert while at work, eating, or meeting people during the daytime? | Yes=1·00; no=0·00 |
| 17 | How often do you have bowel movements each week? | <3 times per week=1·00; other=0·00 |
| 18 | During the past 12 months, did you have pain or discomfort in your body lasting ≥3 months that interfered with your life? | Yes=1·00; no=0·00 |
| 19 | During the past 12 months, have you developed a frequent cough? | Yes, for ≥3 months=1·00; yes, for <3 months=0·50; no=0·00 |
| 20 | Do you brush your teeth rarely or never, or have false teeth? | Yes=1·00; no=0·00 |
| 21 | Physical activity in the past 12 months, including the usual type and duration of activities in occupational, commuting, domestic, and leisure time-related domains | Lowest quintile stratified by sex=1·00; other=0·00 |
| 22 | During the past 12 months, have you lost weight (≥2·5 kg) despite not trying to intentionally lose weight? | Yes=1·00; no=0·00 |
| 23 | During the past 12 months, did you feel much sadder, or more depressed, than usual for ≥2 weeks? | Yes=1·00; no=0·00 |
| 24 | How is your current general health status? | Poor=1·00; fair=0·50; good=0·25; excellent=0·00 |
| 25 | Body-mass index (kg/m^2^)[*](https://www.thelancet.com/journals/lanpub/article/PIIS2468-2667(20)30113-4/fulltext#tbl1fn1) | <18·5 or ≥28·0=1·00; ≥24·0 and <28·0=0·50; ≥18·5 and <24·0=0·00 |
| 26 | Waist circumference (cm) to hip circumference ratio | ≥0·95 for men or ≥0·90 for women=1·00; ≥0·90 and <0·95 for men or ≥0·85 and <0·90 for women=0·50; <0·90 for men or <0·85 for women=0·00 |
| 27 | Measured heart rate, beats per min | <60 or >100=1·00; ≥60 and ≤100=0·00 |
| 28 | The ratio of forced expiratory volume in 1 s to the forced vital capacity measured to be <0·7 | Yes=1·00; no=0·00 |
